# Intrinsic and extrinsic motivators of attachment under active inference

**DOI:** 10.1371/journal.pone.0193955

**Published:** 2018-04-05

**Authors:** David Cittern, Tobias Nolte, Karl Friston, Abbas Edalat

**Affiliations:** 1 Department of Computing, Imperial College London, London, United Kingdom; 2 Wellcome Trust Centre for Neuroimaging, University College London, London, United Kingdom; 3 Anna Freud Centre, London, United Kingdom; New York University, UNITED STATES

## Abstract

This paper addresses the formation of infant attachment types within the context of active inference: a holistic account of action, perception and learning in the brain. We show how the organised forms of attachment (secure, avoidant and ambivalent) might arise in (Bayesian) infants. Specifically, we show that these distinct forms of attachment emerge from a minimisation of free energy—over interoceptive states relating to internal stress levels—when seeking proximity to caregivers who have a varying impact on these interoceptive states. In line with empirical findings in disrupted patterns of affective communication, we then demonstrate how exteroceptive cues (in the form of caregiver-mediated AMBIANCE affective communication errors, ACE) can result in disorganised forms of attachment in infants of caregivers who consistently increase stress when the infant seeks proximity, but can have an organising (towards ambivalence) effect in infants of inconsistent caregivers. In particular, we differentiate disorganised attachment from avoidance in terms of the high epistemic value of proximity seeking behaviours (resulting from the caregiver’s misleading exteroceptive cues) that preclude the emergence of coherent and organised behavioural policies. Our work, the first to formulate infant attachment in terms of active inference, makes a new testable prediction with regards to the types of affective communication errors that engender ambivalent attachment.

## Introduction

During the early stages of life, an infant is highly dependent on others for survival. Attachment theory posits that each infant is genetically pre-disposed to seek out an emotionally supportive, dependent relationship with a primary caregiver, to whom they turn for comfort and safety during times of stress or perceived threat [1, 2]. The central tenet of attachment theory is that the nature of early dyadic attachment interactions (and in particular the caregiver’s response to emotionally-charged bids for proximity by the infant) lead to particular, distinguishable types of attachment representations. These attachment patterns are thought to reflect an internal working model (attachment schema) that captures the extent to which the infant believes they can rely on the caregiver for assistance in emotion and stress regulation, which is rooted in brain circuitry that is shaped by experience-dependent plasticity and epigenetics [3, 4]. Attachment patterns manifest in different behaviours during times of arousal or dysregulation. Early interactions are thought to fundamentally shape the development of the embodied self and mental models of physiological states [5], and the ensuing attachment schema is generalised to other socially challenging and emotionally charged situations or relationships encountered later in life [6, 7]. Furthermore, certain types of suboptimal attachment experience have been linked to a predisposition for the development of various psychological disturbances [8–12].

### The nature of attachment

Attachment theory grew out of the work of John Bowlby, who began to study early mother-child relationships (and in particular the effects on the child of maternal deprivation and separation) in the 1940s. Bowlby theorised that it was the nature of the infant’s cumulative early experience with their caregiver that was key to understanding attachment and subsequent emotional development. He developed attachment theory over the following decades [1, 2, 13–17]; framing it as a lasting interpersonal connectedness—at the representational level—that had a biological, evolutionary basis. He took the view that each child was genetically predisposed to form an attachment with a primary caregiver, to whom they would seek proximity in times of distress, fear or perceived danger. According to Bowlby, the attachment system (a result of natural selection) interacts with other behavioural systems (fear, exploratory, caregiving), whose collective primary goal is experience of felt security in, and survival of, the infant. He hypothesised that the attachment behavioural system is activated in response to both internal (e.g. pain, hunger) and external (e.g. being threatened or endangered) events, triggering proximity seeking behaviours that in turn activate the caregiving behavioural system (in the caregiver). This ensures that the infant and caregiver are jointly predisposed to seek and maintain proximity with each other. Central to this attachment behavioural system was the concept of Internal Working Model (IWM): a representation of the attachment relationship and its participants that the infant builds based on its particular experiences. This IWM is used to generate expectations and predictions about future attachment-related experiences and caregiving behaviour, which are used by the infant to make decisions about how they should act to achieve their goal of felt security.

### Empirical classification

Application of a controlled laboratory procedure called the Infant Strange Situation (ISS)—designed to activate the infant’s attachment system by way of stranger interaction and caregiver separation-reunions in an unfamiliar environment—has uncovered distinct attachment types [18, 19]. These attachment types are secure, along with three insecure (avoidant, ambivalent and disorganised) types; each of which is associated with a different pattern of caregiving in the home environment. While attachment theory focuses on the impact of the primary caregiver on the emergence of infant attachment style—and does not consider the potential impact of broader societal factors, secondary caregivers or peers—research suggests that the fundamental principles and individual differences it describes have a cross-cultural universality (although with differences in distributions of attachment types across societies) [20].

During the ISS, secure infants are characterised by an ardent desire to explore their environment in the presence of the caregiver, distress on separation and proximity seeking on reunion, and are quick to be consoled and return to exploration once they achieve proximity. In other words, secure infants are characterised by effective use of the caregiver as a secure base for exploring their environment. A broad body of research has found caregiving sensitivity (defined as being alerted by the infant’s signals, interpreting them accurately, and responding appropriately and promptly) to be a strong predictor of the emergence of secure infant attachment [19, 21–23]. Additional work has highlighted the ability of these caregivers to treat their child as an intentional agent [24].

In contrast, avoidant infants continue to explore the environment when the caregiver leaves the room, and avoid them completely on reunion. Although these infants display little outward distress on separation, studies have found increased heart rate [25–27], decreased heart rate variability [28, 29] and increased cortisol [26] (although see [30, 31]) in response to the procedure. This suggests that avoidant infants experience separation anxiety but attempt to regulate or repress internal stress and emotion themselves. Caregivers of avoidant infants have been found to be dismissing or rejecting of the infant’s bids for connection, and to be more emotionally unavailable and distant [32, p.150].

Ambivalent infants exhibit a far smaller degree of environment exploration compared to the secure and avoidant infants; instead tending to remain preoccupied with the caregiver’s proximity throughout the ISS. On reunion, ambivalent infants tend to seek proximity but resist attempts by the caregiver to soothe their distress. Compared to the secure infants, who seek proximity but accept comfort from the caregiver, ambivalent infants have been observed to take longer to console and return to exploring their environment [19]; suggesting that this resistant (guarded) behaviour might serve to dampen the effect that the caregiver has on their internal states. An ambivalent infant attachment style has been found to correlate with inconsistent caregiving that fluctuates between under- and over-involvement [32, p.150], and (as will be seen) disrupted patterns of affective communication.

The secure, avoidant and ambivalent attachment types are typically considered to be organised forms of attachment, in that they manifest in coherent and consistent behavioural strategies that are thought to result from adaptations to the (attachment-related) behaviour of their particular caregiver. A relatively small number of infants in the ISS do not fit into one of these three organised classifications and instead appear to lack a coherent strategy for their attachment behaviour [33]. These infants—classified as disorganised—display bizarre or contradictory behaviours when reunited with the caregiver; namely, behaviours that are displayed without an immediately obvious explanation and often amidst behaviours associated with the organised strategies. These include sequential or simultaneous displays of contradictory behaviour (e.g. strong proximity seeking followed by strong avoidance), asymmetrical and mistimed movements and expressions (e.g. sudden jerky movement), and direct displays of apprehension or fear towards the caregiver (e.g. stifled screaming) [34, 35, p.25]. Disorganised infants—considered insecure since they do not effectively use their caregiver as a secure base—are described as either lacking a coherent strategy altogether, or being inclined towards a particular secondary organised strategy (secure, avoidant or ambivalent) that they are unable to fully realise. Infant disorganisation has been linked to caregiver maltreatment [36, 37] and frightened or frightening caregiving behaviour [38–41]. These frightened/frightening behaviours [42] are thought to lead to disorganisation as a result of an unsolvable dilemma; in that the caregiver (i.e. the secure base from whom the infant seeks comfort) also comes to be associated with being a source of fear (“fear without solution”) [43].

#### Disrupted affective communication

More recently, evidence has emerged to suggest a role for atypical and disrupted patterns of affective communication in the formation of both ambivalent and disorganised infant attachment types. Building on the frightened/frightening hypothesis on the origins of disorganisation, Lyons-Ruth et al. considered a wider variety of caregiving behaviours and communication patterns (encompassing frightened/frightening behaviour) in terms of their overall ability to moderate the infant’s distress [44]. Under this view, competing parental attachment tendencies in the caregiver (e.g. drives to simultaneously invite and reject the infant) are thought to manifest in patterns of disrupted affective communication and misattunement, which are proposed to be ineffective in regulating the infant’s internal state and compromise their ability to organise a strategy for attachment. Caregiver disrupted affective communication is coded with the Atypical Maternal Behaviour Instrument for Assessment and Classification (AMBIANCE) scale [45, 46, Appendix G], which has five dimensions: ACE, Role/Boundary Confusion, Fearful/Disoriented Behaviours, Intrusiveness/Negativity, and Withdrawal.

Elevated rates of ACEs (i.e., affective communication errors) have been found in mothers of infants classified as either ambivalent [46] or disorganised [47]. Maternal ACEs have also been found to correlate with an increased prevalence of disorganised infant behaviours in general (i.e., independent of final overall classification), along with increased resistance in both organised infants and a subset of disorganised infants (those tending towards avoidance and/or resistance rather than proximity seeking) [44]. In [44], ACE was the only dimension of the AMBIANCE scale that differentiated mothers of organised and disorganised infants. Furthermore, increased levels of disrupted affective communication (a broader construct including ACE) have been found in mothers of disorganised versus organised infants [48]; in a subset of disorganised infants (tending towards avoidance and/or resistance rather than proximity seeking) [48]; and in mothers of ambivalent and disorganised infants compared to secure and avoidant [49]. These findings support the hypothesis, pointing to heightened occurrence of disrupted affective communication (and ACEs in particular) in mothers of disorganised (especially those tending towards avoidance and/or resistance) and ambivalent infants.

### Significance

#### Stability and transmission of attachment types

Studies probing attachment working models in adults (e.g. using the Adult Attachment Interview, AAI [50, 51]) suggest a degree of stability in attachment type from infancy into adulthood (see [32], p.155 for a summary), with secure to insecure changes typically linked to adverse life experiences or trauma (e.g. [52]), and insecure to secure transformations also possible (e.g. [53]). Furthermore, it appears that a caregiver’s prenatal adult attachment type predicts their infant’s attachment type with some accuracy [54]. Overall, this suggests a tendency towards intergenerational transmission of attachment types (although this transmission is by no means inevitable, especially given intervention).

#### Attachment and psychological health

Attachment experiences and representations are increasingly recognised as important for understanding and promoting psychological health. While secure attachment is thought to be associated with mental resilience and relatively quick recovery from stress, it has been argued that insecure forms of attachment (associated with a wide range of psychopathology, including depression, clinical anxiety, and various personality disorders) can be viewed as a general vulnerability to (although not necessarily a sufficient cause of) mental disorders, with particular symptomatology influenced by other factors including genetic and environmental [55]. This general link between attachment insecurity and psychopathology is thought to be mediated by dysfunctional beliefs about the self and others, disruptions in the development of capacities for regulation (including self-regulation) of emotion, and ensuing problems in interpersonal relationships [55].

From a clinical perspective, disorganised forms of attachment are particularly significant. A prominent theoretical account of Borderline Personality Disorder (BPD, a disorder characterised by affective instability and difficulties of interpersonal exchange [56, 57]) posits a developmental basis for the disorder in early disorganised forms of attachment experience [8, 9], which is supported by longitudinal evidence [10]. BPD shows a strong intergenerational effect (see discussion in [58]), and BPD mothers (who have a higher prevalence of disorganised AAI states of mind [59, 60]) show an increased tendency for infant-directed behaviour that leads to insecure and disorganised forms of attachment (including frightened behaviours and ACEs [61]), and a decreased tendency towards positive and affiliative behaviours; both leading to epistemic hypervigilance as a form of compromised social information processing [62, 63].

Disorganised attachment has also been linked to dissociation, which is typically defined as a deficiency in the integration of memory, consciousness and identity that manifests as either a lack of attention to the external environment or sudden breaks in the continuity of thought or behaviour (of which the individual is unaware) [12]. Dissociative states of mind are associated with both individuals with BPD and those classified as unresolved (disorganised) in the AAI, and disorganised infants display many behaviours within the context of attachment that are similar to those indicative of dissociation in adults [12, 64]. Indeed, it has been proposed that these behaviours might be the first instance of dissociative reactions during life, and that early disorganised attachment experiences increase the vulnerability for dissociative reactions to other traumas later in life [11, 12].

### Computational models of attachment

Recent studies have started to characterise dyadic attachment using computational models. In contrast to our work, these studies do not tend to consider the (clinically most important) disorganised forms of attachment, nor the body of research on disrupted patterns of affective communication (and in particular the misleading and ambiguous signalling that we will consider here) and insecurity. In [65] a dynamical systems model of infant attachment was presented, with the role of the caregiver considered to be a regulator of the infant’s internal physiological (opioid-modulated and arousal) state, which drives exploratory and attachment behaviour. The authors proposed that secure, avoidant and ambivalent infants could be characterised by different levels of sensitivity to opioids and arousal. An alternative dynamical systems model of organised attachment types is presented in [66], which defines infant anxiety in terms of a variable representing the insensitivity of the caregiver to the infant’s needs, infant-specific parameters (governing how they return to baseline following a stressful episode and their emotional stability), and the emotional distance between the infant and caregiver. Emotional distance, which describes proximity seeking behaviour, is governed by parameters defining the caregiver’s inconsistency and insensitivity and the infant’s intrinsic curiosity, along with the infant’s current anxiety level. [66] formulates attachment (although not specific types) in terms of a feedback system grounded in control theory. The system, representing the infant, amplifies externally induced distress in the absence of the caregiver. The controller, representing the caregiver, regulates infant distress by way of three gains (corresponding to the regulatory ability of the caregiver, the healthiness of the past relationship, and their consistency).

The goal-based cognitive agent architectures in [67, 68] consider organised attachment types arising from exploratory, fear and security systems. The infant explores their environment with a proximity safe-range distance that is adjusted based upon the caregiver’s delay in responding to infant signalling. The caregiver will only respond to the infant when they signal above some particular threshold. Simulations revealed a critical point for this threshold—that determines whether the dyad develops into a secure or insecure style. Avoidant attachment is accounted for in two ways: either as a result of an ethological displacement-like inhibition of the security goal, or in response to deliberative recall of memories of previous rejection.

In developmental robotics, the attachment secure base and dyadic arousal regulation paradigms have been studied as drives for a robot’s exploration and learning in a novel environment [69, 70]. In this setting, the robot has a single goal, which is to learn the best model of its environment, while balancing an internal measure of arousal (defined by the degree to which it remembers and recalls percepts) that dictates its behaviour. In particular, when the arousal level is low, the robot continues to explore and learn, but when arousal is too high it seeks comfort from a human attachment figure (resuming exploration once its arousal drops below its tolerable threshold). The arousal-based neurocognitive model in [71]—a synthesis of the work in [67] and [69]—accounts for basic infant attachment behaviour and physiology in an ISS-like separation and reunion scenario (following episodes of learning based on secure-base exploration and proximity seeking). Under this model, arousal levels are driven by a measure of novelty during exploration (representing the degree to which the infant is overwhelmed by environmental perceptions) and an adaptive safe-range distance (as in [67]), along with fear circuitry activation on retrieval of memories of previously hostile caregiving. As described in computationally informed conceptualisation, according to [72] the ISS simultaneously gives rise to activation of a number of prototype emotion systems such as fear and anger.

Several studies have also attempted to model aspects of various psychotherapeutic processes relevant to attachment. Attachment schemas and prototypes have been considered within the context of strong patterns in a Hopfield network, which have been proposed to provide a conceptual model of both the acquisition of—and psychotherapeutic-driven changes to—attachment type [73, 74]. [75] considers mentalization-based psychotherapies, and how changes in activation in attachment-related brain areas might underlie a shift towards more deliberative forms of decision making. [76] manipulates reward in a multi-agent reinforcement learning setting to describe the application of Self-Attachment therapy [77, 78], the hypothesised neurobiological effects of which are modelled in [79] and [80]. Finally, a broader self (with others) representational framework with an approximate Bayesian inference architecture has been proposed by [81, 82]. Under the framework, the self models beliefs about the traits of both the self and other, and uses this to model the beliefs that the other has about the traits of self and other. These beliefs about self and other are actively inferred in a mentalization-like manner. The capacity to understand that other people’s actions are caused by their beliefs begins to develop around ages three to four [83], which is older than the strange situation-aged infants that we consider here.

#### Game theoretic formulations

A variety of game theoretic models of attachment are presented in [66], showing how secure, avoidant and ambivalent forms of attachment might emerge as equilibrium decision choices. The authors begin by considering a single player game (i.e. a decision theoretic model) of an infant’s choice as to whether to seek out or avoid a caregiver who might either attend to or ignore them. The infant is assumed to have stress above some tolerable level, and the payoffs correspond to changes in stress (i.e. increases or decreases). When the model includes a guarded (resistant) form of proximity seeking—that dampens the effect of attention/rejection on decreases/increases in infant stress—the authors show how proximity seeking, guarded proximity seeking or avoidance behaviour (corresponding to the three organised forms of attachment) emerge as optimal responses to caregivers with responsiveness profiles that fall into three distinct regions. This single player game is then extended to consider payoffs for the caregiver, given these joint-action outcomes. This enabled the authors to show how Nash equilibria corresponding to secure, avoidant and ambivalent attachment relationships emerge according to particular payoff configurations.

All of the above models are normative, in the sense that they provide a formal description of attachment behaviour; usually, under an optimisation assumption. In other words, they assume the existence of some (stress-related) objective function that can be optimised with appropriate dynamics, behaviours or choices. Our approach is based upon a generic normative theory called active inference, which also provides a biologically plausible process theory for how the underlying computations and dynamics might be implemented in the brain. We apply this generic formalism using the payoff structure for different proximity seeking behaviours, established by the game theoretic models above.

#### An active inference formulation

In this paper, we use a generic formulation of intrinsically and extrinsically motivated behaviour (active inference under the free energy principle) that is predicated on the game theoretic payoffs above. We pursue the hypothesis that different attachment patterns emerge as (Bayes) optimal responses to different experiences of a caregiver. Active inference casts everything in terms of beliefs about states of the world (and body) and, crucially, the consequences of different behaviours under a generative model of dyadic interactions. This generative model corresponds to the Internal Working Model introduced by Bowlby but cast in formal (Bayesian) terms.

In what follows, we will adopt the game theoretic formulation of differential payoffs for attachment behaviour and associate them with the prior preferences of a generative model. We will see in the next section that these payoffs correspond to prior beliefs about the likelihood of different outcomes and scaffold the *extrinsic value* of a response or policy. Crucially, in active inference, this extrinsic value is supplemented with an *epistemic value*. Epistemic value drives exploration of behaviour in order to reduce uncertainty about states of the environment, and corresponds to the intrinsic motivation for exploratory behaviour in developmental neurorobotics.

Here, we focus on the decision-theoretic formulation with a guarded (resistant) request for comfort, as seen in ambivalent infants [66]. This is used as a starting point for our active inference formulation of attachment. This formulation calls for a quantitative specification of allowable actions and their consequences. In detail, we will assume the probability that the caregiver attends is 0 ≤ *q* ≤ 1. When the infant seeks comfort (i.e. approaches) and the caregiver attends to them, the payoff to the infant is *g*. On the other hand, if the infant seeks proximity but the caregiver ignores them, the payoff is −*m*. If the infant is stressed by this rejection then *m* > 0; whereas if they are comforted by proximity to the caregiver (even though the caregiver ignores them) then *m* < 0. In this second case, it is assumed that −*m* < *g*. In other words, if the infant is ignored, they receive less comfort than if the caregiver attends. If the infant does not go to the caregiver for comfort then they receive no comfort, regardless of what the caregiver does (i.e. a payoff of zero). Finally, if the infant seeks proximity to the caregiver in a guarded fashion then outcomes are parameterised by *h* and *n*. It is assumed that 0 < *h* < *g*; i.e., comfort received from guarded proximity seeking is less than for comfort seeking, but more than for avoiding. As for *m*, there are two cases for the sign of *n*: if *n* < 0 then the infant receives comfort from being near the caregiver, even if the caregiver ignores them (in this case it is assumed that −*g* < *m* < *n* < 0). If *n* > 0 then the infant is stressed by the caregiver ignoring them (in this case it is assumed that 0 < *n* < *m*).

We consider here the cases of *q* for 0 < *n* < *m*, and either *h* > *gn*/*m* or *h* < *gn*/*m* (*h* > *gn*/*m* allows for the selection of the three actions, and thus three attachment types, as optimal responses to the caregiver with a known *q* under game theoretic assumptions [66]). In what follows, we briefly review the active inference formulation; paying particular attention to the role of extrinsic and epistemic value in action selection. In subsequent sections we will use the payoffs above to examine how extrinsic, exploitative, goal-seeking behaviour interacts with epistemic, exploratory, novelty seeking behaviour to produce distinct attachment behaviours that bear a remarkable similarity to those observed empirically.

## Materials and methods

The free energy principle is a theory of self organisation which suggests that biological systems (such as the brain) resist a tendency to disorder by restricting themselves to a small number of physiological and sensory states that they a priori prefer to occupy [84]. The theory argues that the only tractable way the brain can restrict itself to preferred states is by minimising a quantity called free energy. This quantity provides an upper bound on a measure of surprise (that increases as a function of the improbability or undesirability of encountered states). According to the theory, action, perception and learning are all fundamentally driven by a minimisation of free energy, with the resulting process (describing loops of interaction between an agent and its environment) referred to as Active Inference.

We follow the mathematical formulation outlined in [85] and [86] based on a partially observable Markov decision process. This formulation has been used in numerous simulations of optimal (and suboptimal) behaviour; ranging from choice behaviour in economic games to scene construction and saccadic eye movements [87, 88]. The equations below may look complicated; however, they follow from standard results for belief updating and variational learning, in the context of Markov decision processes. In brief, this formulation considers a finite set *O* of *W* observations (or observable outcomes), a finite set *S* of *J* discrete hidden states and a finite set Ω of *L* discrete actions. We denote a finite sequence over time of observations in *O*, or hidden states in *S*, or actions in Ω, respectively, by $\tilde{o}$, $\tilde{s}$ and $\tilde{a}$, where the length of the sequence is made clear in the situation. A generative process *R* generating outcomes from hidden states—up to the current time *t*—can then be specified probabilistically:

$$R\left(\tilde{o},\tilde{s},\tilde{a}\right)=Pr\left(\left\{o_{0},\ldots ,o_{t}\right\}=\tilde{o},\left\{s_{0},\ldots ,s_{t}\right\}=\tilde{s},\left\{a_{0},\ldots ,a_{t}\right\}=\tilde{a}\right),$$ (1)

where *o*_*i*_ ∈ *O*, *s*_*i*_ ∈ *S* and *a*_*i*_ ∈ Ω for 0 ≤ *i* ≤ *t*. The agent is assumed to have an internal working model of this generative process, called their “generative model” (i.e. an internal model of how hidden causes generate sensory data). This is the formal homologue of the Internal Working Model (IWM) above. The agent’s generative model over finite sequences $\tilde{o}$ of observations, finite sequences $\tilde{s}$ of hidden states and finite sequences $\tilde{u}$ of control states is:

$$P\left(\tilde{o},\tilde{s},\tilde{u}\right)=Pr\left(\left\{o_{0},\ldots ,o_{T}\right\}=\tilde{o},\left\{s_{0},\ldots ,s_{T}\right\}=\tilde{s},\left\{u_{0},\ldots ,u_{T}\right\}=\tilde{u}\right)$$ (2)

which (unlike the generative process) includes beliefs about future states up to time *T* > *t*. Under the generative model, actions (*a*, a variable that acts on the generative process) are distinguished from control states (*u*, the corresponding *random* variable in the generative model). Because control states are random variables, they are inferred. Action is then sampled from the resulting beliefs about control.

Policies *π* ∈ *U*^*T*−*t*+1^ index sequences of future control states $\left(\tilde{u}|\pi \right)=\left(u_{t},\ldots ,u_{T}\right)$ and thus there are *K* = |*U*|^*T*−*t*+1^ policies available, where *U* is the set of all control states, and |*U*| is the number of available control states. It is assumed that the agent has an approximate posterior distribution *Q* over hidden and control states:

$$Q\left(\tilde{s},\tilde{u}\right)=Pr\left(\left\{s_{0},\ldots ,s_{T}\right\}=\tilde{s},\left\{u_{0},\ldots ,u_{T}\right\}=\tilde{u}\right)$$ (3)

In other words, it has beliefs about both the states of the world and the policies which it is currently pursuing. These beliefs are parameterised by expectations: $\left(\overset{⌢}{s},\overset{⌢}{\pi }\right)$, where $\overset{⌢}{s}\in {\left[0,1\right]}^{J}$ is a *J* × 1 probability vector of expected states, and $\overset{⌢}{\pi }\in {\left[0,1\right]}^{K}$ is a *K* × 1 vector of policy expectations. The agent is further assumed to have a prior distribution specifying the utility (preference) of each outcome at time *τ* > *t*:

$$P\left(o_{\tau }\right)=C_{\tau }$$ (4)

These (prior) preferences correspond to the extrinsic motivation of the preceding section.

The free energy principle argues that agents aspire to minimise a quantity called surprise $-\text{ln}\;P(\tilde{o})$. According to the theory, the only tractable way to do this is by minimising a free energy functional *F* of the approximate posterior distribution:

$$\begin{array}{c} \begin{array}{cc} F(\tilde{o},\overset{⌢}{s},\overset{⌢}{\pi }) & =E_{Q}[-\text{ln}\;P\left(\tilde{o},\tilde{s},\tilde{u}\right)-H\left[Q\left(\tilde{s},\tilde{u}\right)\right]\\ & =-\text{ln}\;P\left(\tilde{o}\right)+KL[Q\left(\tilde{s},\tilde{u}\right)||P\left(\tilde{s},\tilde{u}|\tilde{o}\right)] \end{array} \end{array}$$ (5)

Here $H\left[P\left(x\right)\right]=E_{P(x)}\left[-\text{ln}\;P\left(x\right)\right]$ denotes entropy, and $KL\left[Q\left(x\right)||P\left(x\right)\right]=E_{Q(x)}\left[\text{ln}\;Q\left(x\right)-\text{ln}\;P\left(x\right)\right]$ is a Kullback-Leibler divergence. Crucially, since the KL divergence in Eq 5 cannot be less than zero, when free energy is minimised the approximate posterior distribution approximates the true posterior and free energy becomes an upper bound on surprise [89]. In short, minimising free energy entails Bayesian inference about the hidden states of the world causing data. In virtue of the fact that surprise is also known as (negative log) evidence, free energy minimisation is also referred to as self-evidencing [90].

To derive the updates that minimise free energy, we assume the following factorisation for the generative model:

$$P\left(\tilde{o},\tilde{x}|\tilde{a}\right)=P\left(\tilde{o}|\tilde{s},A\right)P\left(\tilde{s}|\tilde{a},B,D\right)P\left(\tilde{u}|\gamma \right)P\left(\gamma |\alpha ,\beta \right)P\left(A|\theta \right)P\left(B|\phi \right)P\left(D|\xi \right)$$ (6)

where the unknown quantities are summarised with $\tilde{x}=\tilde{s},\tilde{u},\gamma ,A,B,D$.

The first factor $P\left(\tilde{o}|\tilde{s},A\right)=P\left(o_{0}|s_{0},A\right)P\left(o_{1}|s_{1},A\right)...P\left(o_{t}|s_{t},A\right)$, defining observations given hidden states, is encoded in matrix form (such that column *j* of *A*, i.e. *A*_•*j*_, encodes the likelihood of observations given hidden state *j*):

$$P\left(o_{t}=i|s_{t}=j,A\right)=A_{ij}$$ (7)

The second factor $P\left(\tilde{s}|\tilde{a}\right)=P\left(s_{t}|s_{t-1},a_{t},B\right)...P\left(s_{1}|s_{0},a_{1},B\right)P\left(s_{0}|D\right)$ defines hidden state transitions (and the initial hidden state) under the assumption that the agent knows their past actions, and is encoded in matrix form as:

$$P\left(s_{t+1}=i|s_{t}=j,u_{t},B\right)=B{\left(u_{t}\right)}_{ij}$$ (8)

$$P\left(s_{0}=i|D\right)=D_{i}$$ (9)

The third factor $P\left(\tilde{u}|\gamma \right)=\sigma \left(\gamma \cdot Q\right)$ expresses beliefs about sequences of control states (i.e. policies), with *σ* a softmax function. Here, **Q** is a *K* × 1 vector containing the expected negative free energy of each policy at the current time *t*, so that **Q**(*π*) scores the negative free energy expected under each policy *π*:

$$Q(\pi )=\sum_{\tau =t+1}^{T}E_{Q(o_{\tau },s_{\tau }|\pi )}\left[\text{ln}\;P\left(o_{\tau },s_{\tau }\right)\right]+H\left[Q\left(s_{\tau }|\pi \right)\right]$$ (10)

where $Q\left(o_{\tau },s_{\tau }|\pi \right)=P\left(o_{\tau }|s_{\tau }\right)Q\left(s_{\tau }|\pi \right)=E_{Q(s_{t})}\left[P\left(o_{\tau },s_{\tau }|s_{t},\pi \right)\right]$ is a posterior predictive distribution over future states and outcomes. It is this factor (expectations over policies) that endows active inference with extrinsic and epistemic aspects in virtue of the ways in which expected free energy can be decomposed into key components. We will return to this in the last section.

The fourth factor *P*(*γ*|*α*, *β*) expresses a prior over precision *γ* (encoding confidence in prior beliefs), which is assumed to have a gamma distribution with shape and rate parameters *α* and *β*:

P(γ|α,β)=Gamma(α,β) (11)

The fifth factor *P*(*A*|*θ*) is a Dirichlet prior (with concentration parameters *θ*) over the multinomial distributions *A*_•*j*_ (encoding the likelihood of observations given hidden state *j*):

$$P\left(A_{•j}|\theta \right)=Dirichlet\left(\theta_{•j}\right)$$ (12)

Similarly, the sixth factor *P*(*B*|*ϕ*) is a Dirichlet prior (with concentration parameters *ϕ*) over the multinomial distributions *B*(*u*)_•*j*_ encoding the likelihood of hidden states at *t* + 1 given that the hidden state at time *t* is *j*:

$$P\left(B{\left(u\right)}_{•j}|\phi \left(u\right)\right)=Dirichlet\left(\phi {\left(u\right)}_{•j}\right)$$ (13)

The final factor *P*(*D*|*ξ*) is a Dirichlet prior (with concentration parameters *ξ*) over the multinomial distribution encoding the initial hidden state:

P(D|ξ)=Dirichlet(ξ) (14)

For the approximate posterior *Q*, a simpler factorisation is assumed that renders the minimisation of free energy tractable (technically, this is known as a mean field assumption):

$$Q(\tilde{x}|\overset{⌢}{x})=Q\left(s_{0}|{\overset{⌢}{s}}_{0}\right)\ldots Q\left(s_{T}|{\overset{⌢}{s}}_{T}\right)Q\left(u_{t},\ldots ,u_{T}|\overset{⌢}{\pi }\right)Q\left(\gamma |\overset{⌢}{\gamma }\right)Q\left(A|\overset{⌢}{\theta }\right)Q\left(B|\overset{⌢}{\phi }\right)Q\left(D|\overset{⌢}{\xi }\right)$$ (15)

The approximate posterior is parameterised in terms of its expectations $\overset{⌢}{x}=\left(\overset{⌢}{s},\overset{⌢}{\pi },\overset{⌢}{\gamma },\overset{⌢}{\theta },\overset{⌢}{\phi },\overset{⌢}{\xi }\right)$, where:

$$Q\left(\gamma |\overset{⌢}{\gamma }\right)=Gamma\left(\alpha ,\overset{⌢}{\beta }=\alpha /\overset{⌢}{\gamma }\right)$$ (16)

$$Q\left(A|\overset{⌢}{\theta }\right)=Dirichlet\left(\overset{⌢}{\theta }\right)$$ (17)

$$Q\left(B|\overset{⌢}{\phi }\right)=Dirichlet\left(\overset{⌢}{\phi }\right)$$ (18)

$$Q\left(D|\overset{⌢}{\xi }\right)=Dirichlet\left(\overset{⌢}{\xi }\right)$$ (19)

Given these factorisations, it can be shown that the variational updates of the expectations that minimise free energy are given by [85, 86]:

$${\overset{⌢}{s}}_{t}=\{\begin{array}{cc} \sigma (\overset{⌢}{A}\cdot o_{t}+\overset{⌢}{D}) & \;\text{if}\;t=1\\ \sigma (\overset{⌢}{A}\cdot o_{t}+\overset{⌢}{B}\left(a_{t-1}\right){\overset{⌢}{s}}_{t-1}) & \;\text{otherwise} \end{array}$$ (20)

$$\overset{⌢}{\pi }=\sigma \left(\overset{⌢}{\gamma }\cdot Q\right)$$ (21)

$$\overset{⌢}{\gamma }=\alpha /\left(\beta -Q\cdot \overset{⌢}{\pi }\right)$$ (22)

$${\overset{⌢}{\theta }}_{ij}=\theta_{ij}+\sum_{t=1}^{T}{o_{t}}_{i}{{\overset{⌢}{s}}_{t}}_{j}$$ (23)

$$\overset{⌢}{\phi }{\left(u\right)}_{ij}=\phi {\left(u\right)}_{ij}+\sum_{t=2}^{T}\left[u=a_{t-1}\right]\cdot {{\overset{⌢}{s}}_{t}}_{i}{{\overset{⌢}{s}}_{t-1}}_{j}$$ (24)

$$\overset{⌢}{\xi }=\xi +{\overset{⌢}{s}}_{1}$$ (25)

For ${\overset{⌢}{A}}_{ij}=E_{Q}\left[\text{ln}\;A_{ij}\right]=\psi \left({\overset{⌢}{\theta }}_{ij}\right)-\psi \left(\sum_{i}{\overset{⌢}{\theta }}_{ij}\right)$, ${\overset{⌢}{B}}_{ij}=E_{Q}\left[\text{ln}\;B_{ij}\right]=\psi \left({\overset{⌢}{\phi }}_{ij}\right)-\psi \left(\sum_{i}{\overset{⌢}{\phi }}_{ij}\right)$ and ${\overset{⌢}{D}}_{i}=E_{Q}\left[\text{ln}\;D_{i}\right]=\psi \left({\overset{⌢}{\xi }}_{i}\right)-\psi \left(\sum_{i}{\overset{⌢}{\xi }}_{i}\right)$, with *ψ* the digamma function, and the Iverson brackets [⋅] returning one if the expression is true and zero otherwise.

The first three of these updates (Eqs 20–22) are inference updates, and are iterated until convergence (or *N* times) after each new observation is sampled. Briefly, following an observation, the agent iterates these inference updates before selecting an action that minimises expected free energy (sampled from $\overset{⌢}{\pi }$). On performing this action, the environment transitions to a new hidden state and provides the agent with a new observation. These perception and action steps repeat until the end of the trial or episode. The variational updates involved in perception (inference about the hidden state, Eq 20) have been associated with computations in the prefrontal cortex, while the updates underlying action selection (Eq 21) have been linked with activity in the striatum, and the expected precision (Eq 22) has been associated with dopaminergic signals from the ventral tegmental area and substantia nigra [85]. The final three updates (Eqs 23–25) are Hebbian-like learning updates with implicit learning rates determined by the amount of prior experience, and are typically performed following each length-T trial (episode) [86].

This concludes our formal description of active inference for discrete state space (i.e., Markov decision process) models based upon minimising (expected) free energy. In the next section, we describe the generative model (and process) used to simulate attachment behaviour under the prior preferences offered by gain theoretic formulations of payoffs in dyadic interactions with a caregiver.

## Results

The imperative to minimise free energy—the notion that agents act, perceive and learn in order to restrict themselves to some limited number of a priori preferred states—speaks to the challenge facing the infant’s developing brain; directing action, performing emotional appraisal and learning about the characteristics of their attachment caregiver to ensure homeostasis. Thus, using the decision theoretic model outlined above as a starting point, we can formulate a basic model of attachment in terms of free energy minimisation, with an infant who has prior preferences for interoceptive outcomes associated with low stress states. Evidence suggesting that the physiological stress response is related to subjective estimates of uncertainty [91] fits with our use of the free energy principle and implicit active inference; in the sense that this framework inherently involves a ‘drive’ towards the resolution of uncertainty [85]. We begin by considering an infant who only experiences these interoceptive outcomes, before modelling an infant who also receives exteroceptive observations from the caregiver—that are interpreted in terms of cues relating to subsequent behaviour.

We begin by considering an infant who minimises free energy over interoceptive outcomes relating to stress. As outlined above, *q* quantifies the probability that (at any particular time) the caregiver will respond in a way (i.e., attentively and sensitively) that effectively lowers the infant’s internal stress, should they seek proximity. We refer to this as caregiving “responsiveness” (with responsiveness increasing in *q*).

### The generative process and model

We consider an environment for the infant in terms of the actions (and corresponding control states), observations (corresponding to interoceptive states relating to stress levels), and hidden states. The control states are Seek (*U*_1_, corresponding to the ‘Go’ approach action in the game theoretic models), Guarded Seek (*U*_2_, corresponding to the ‘Half Go’ guarded approach action), and Avoid (*U*_3_, corresponding to ‘Don’t Go’). The corresponding actions are Ω_*i*_ = *U*_*i*_, and we assume that the infant must perform one of these actions at each time step or exchange with the caregiver.

Initially, the observations for the infant are interoceptive outcomes *I* = {*I*_1_, *I*_2_, *I*_3_, *I*_4_, *I*_5_} generated by hidden states. Later, we will consider exteroceptive observations generated by the caregiver. Interoceptive observations are assumed to accurately reflect internal states, and we will ignore any individual variability with regards to awareness of these interoceptive signals [92]. The preference distribution over interoceptive outcomes corresponds to the payoffs in the decision theoretic model, which represent the amount of comfort or stress reduction received by the infant when the caregiver either attends to or ignores them. In particular, *I*_1_ represents the payoff *g*, *I*_2_ is *h*, *I*_3_ is −*m*, *I*_4_ is −*n*, and *I*_5_ is 0. In other words, the payoffs (*g*, *h*, *m* and *n*) parameterise preferences about outcomes.

There are two hidden states modelling caregiving: *X* = {*X*_1_ = Attend, *X*_2_ = Ignore}. These correspond to the caregiver’s regulatory stance towards the infant and determine interoceptive outcomes. For both Attend and Ignore, action Ω_3_ (Avoid) maps to observation *I*_5_, which is the internal state in which there is no change in stress. For Attend caregiving, action Ω_1_ (Seek) maps to observation *I*_1_ (reduction in stress of *g*), and action Ω_2_ (Guarded Seek) maps to observation *I*_2_ (reduction in stress of *h* with *g* > *h* > 0). On the other hand, when the caregiver chooses to Ignore, action Ω_1_ (Seek) maps to observation *I*_3_ (stress increase of *m* relative to the previous time step), and action Ω_2_ (Guarded Seek) maps to observation *I*_4_ (stress increase of *n* with 0 < *n* < *m*).

The complete set of states generating outcomes (in this formulation) corresponds to all combinations of the three control states and the two caregiving behaviours *S* = *U* ⊗ *X*, where ⊗ is the Kronecker tensor product. Although we call these “hidden states”, only states resulting in the interoceptive observation *I*_5_ cannot be determined with certainty based on the observation (the remaining states are fully observable). The hidden state transition probabilities (generative process) are given by:

$$R\left(s_{t+1}|s_{t},a_{t}\right)=G\left(a_{t}\right)$$ (26)

where:

$$G{\left(a_{t}=\Omega_{w}\in \Omega \right)}_{ij}=\{\begin{array}{cc} q & \;\text{if}\;i=2w-1\\ \left(1-q\right) & \;\text{if}\;i=2w\\ 0 & \;\text{otherwise} \end{array}$$ (27)

Current and transitory hidden states are indexed incrementally in columns and rows of *G*(*a*_*t*_), respectively, so that (for example) element *G*(Ω_2_)_13_ contains the probability of transitioning from *S*_3_ to *S*_1_ dependent on Ω_2_. Note that all hidden states can be entered into or left within the time horizon. We assume the homologous form for the generative model.

In the associated generative model, we have created hidden states from combinations of the caregiver’s response and the infant’s behaviour. Strictly speaking, hidden states should not be conflated with control states (because control states determine transitions among hidden states). This means that the hidden state homologues of control states can be regarded as the consequences of the associated action (i.e., proximate, near and distant to the caregiver, following Seek, Guarded seek and Avoid).

For further simplicity, we consider hidden states to represent states for which the infant’s stress levels are (arbitrarily) above some tolerable threshold (i.e. states in which their attachment system is activated). We do not explicitly consider the return to a baseline state (in which the infant’s attachment system is deactivated), since the addition of such a state would require us to define additional transition probabilities. Empirical studies measuring cortisol [26, 30, 31] and heart rate [25–29] during the ISS (i.e. with controlled high caregiving responsiveness) consistently show a more rapid return to baseline for secure infants following the final reunion episode; however, data on relative times to return to baseline for avoidant, ambivalent and disorganised infants is (on the whole) currently inconclusive. Moreover, there is currently no such data for interactions in which caregiving behaviour is uncontrolled. Thus, a return to baseline states (along with a fuller realisation of the secure-base exploration paradigm) is left for future work (see Discussion).

We assume that the infant starts each attachment interaction or episode with some distance between themselves and the caregiver (i.e., in the Avoid state), which represents the most typical scenarios (including environment exploration) under which an infant’s attachment system is activated. We additionally assume that caregiving behaviour on each interaction or episode is determined by the probability *q* governing the caregiver’s overall responsiveness, which results in the following initial hidden state distribution (for the generative process):

$$R\left(s_{0}\right)=\sigma {\left(\left[0\;\;0\;\;1\right]⊗\left[q\;\;\left(1-q\right)\right]\right)}^{⊤}$$ (28)

The possible outcomes for the infant are the set containing all elements of the tensor product of control states and interoceptive observations, i.e. *O* = *U* ⊗ *I*. The (generative process) distribution of outcomes (rows) given hidden states (columns) is given by:

$$R\left(o_{t}|s_{t}\right)=[\begin{array}{c} \lambda^{\left(1\right)}\;\;\;\;\\ \;\;\lambda^{\left(2\right)}\;\;\\ \;\;\;\;\lambda^{\left(3\right)} \end{array}]$$ (29)

with:

$$\begin{array}{c} \lambda^{\left(1\right)}=[\begin{array}{c} 1\;\;0\\ 0\;\;0\\ 0\;\;1\\ 0\;\;0\\ 0\;\;0 \end{array}]\lambda^{\left(2\right)}=[\begin{array}{c} 0\;\;0\\ 1\;\;0\\ 0\;\;0\\ 0\;\;1\\ 0\;\;0 \end{array}]\lambda^{\left(3\right)}=[\begin{array}{c} 0\;\;0\\ 0\;\;0\\ 0\;\;0\\ 0\;\;0\\ 1\;\;1 \end{array}] \end{array}$$ (30)

and the remaining elements of this likelihood or observation mapping are zero. The infant’s preferences about outcomes are given by:

$$P\left(o_{\tau }\right)=C=\sigma {\left({\left\{1\right\}}^{1\times L}⊗\left[g\;\;h\;\;-m\;\;-n\;\;0\right]\right)}^{⊤}$$ (31)

That is, the infant is assumed to be indifferent with respect to control outcomes, but not with respect to interoceptive observations. These preferences are motivated in terms of associated stress levels mediated by the neuroendocrine correlates of the behavioural outcomes we have modelled. These include cortisol and related HPA axis feedback loops (cortisol secreted during a stress response) that increase blood sugar, suppress the immune system, and aid in metabolism to facilitate responses to perceived challenge, uncertainty or threat Sustained (chronic) stress levels in the body can lead to high blood pressure and muscle damage [93]. Evidence from animal and human studies furthermore suggests that chronic stress might lead to a variety of effects on the brain, including cell destruction, changes in proportions of cell types, and decreased plasticity [94] in regions including the hippocampus [95–97], medial prefrontal cortex [98, 99] and orbitofrontal cortex [100]. Thus, one can argue for these priors from an evolutionary perspective, under which prior preferences for prolonged and chronic states of stress would be low (i.e. a priori surprising states).

This concludes our description of the generative model—a Markov decision process that is formulated in a way that speaks to established (decision or game theoretic) formulations of proximity seeking behaviour in the setting of attachment theory. In this setting, the rewards or payoff associated with behavioural outcomes have been cast in terms of prior preferences, which we assumed have been endowed genetically. We now use this model to demonstrate the sorts of behaviour different caregivers could elicit.

### Simulations

We begin by simulating active inference in a synthetic infant that interacts with its caregiver defined by various values of *q*: in each case, the infant is assumed to have a perfect generative model of the environment (i.e. they know or have learned the actual value *q*), which allows us to explore the parameter space. We then consider agents that start with no knowledge of caregiver responsiveness and show that, by learning their generative model, distinct behavioural policies corresponding to secure, avoidant and ambivalent attachment emerge from this common starting point. In all simulations we set prior gamma parameters *α* = 250 and *β* = 1, number of variational iterations *N* = 4, and process depth *T* = 4, with results averaged over 100 independent repetitions. Our implementation uses the SPM12 toolkit [101], which contains routines for implementing the discrete free energy minimisation scheme described above. Additional code for the simulations described here is available from the first author on request.

We start by exploring the parameter space for stress changes resulting from infant seeking and caregiver attention (with preference parameter *g*), infant seeking and caregiver ignoring (*m*), infant guarded seeking and caregiver attention (*h*), infant guarded seeking and caregiver ignoring (*n*), and infant avoidance irrespective of caregiving behaviour (preference 0).

Recall that organised (secure, ambivalent and avoidant) forms of attachment are characterised by coordinated behaviours aimed at achieving either proximity or distance from the caregiver in response to attachment need; compared to disorganised attachment that is characterised by contradictory behaviours. Our aim here was to identify interoceptive preferences that result in the three organised forms of attachment (secure, ambivalent and avoidant), with the degree of attachment organisation measured by consistency of action selection (i.e., the proportion of the corresponding action chosen by the infant with different sorts of caregivers).

To conduct this initial analysis, we assume that the infant has a perfect generative model *B* of the generative process *G* governing hidden state transitions. The generative model is:

$$\overset{⌢}{B}\left(u\right)\approx G\left(a_{t}=u\right)=R\left(s_{t+1}|s_{t},a_{t}\right)$$ (32)

which is achieved with the following Dirichlet concentration parameters:

$${\phi (u)}_{ij}=\{\begin{array}{cc} \epsilon & \;\text{if}\;{G(u)}_{ij}=0\\ \delta \;{G(u)}_{ij} & \;\text{otherwise}\; \end{array}$$ (33)

with *δ* = 10^3^, and *ϵ* = 10^−10^ a small positive number (used to ensure concentration parameters are greater than zero to prevent numerical overflow). Similarly, we begin by assuming that the infant’s model *D* of the true initial hidden state distribution is accurate:

$$\overset{⌢}{D}\approx R\left(s_{0}\right)$$ (34)

according to parameters:

$$\xi_{i}=\{\begin{array}{cc} \epsilon & \;\text{if}\;{R(s_{0})}_{i}=0\\ \delta \;{R(s_{0})}_{i} & \;\text{otherwise}\; \end{array}$$ (35)

Finally, we assume that the infant has a perfect generative model *A* of the generative process governing interoceptive observations given hidden states:

$$\overset{⌢}{A}\approx R\left(o_{t}|s_{t}\right)$$ (36)

parameterised by:

$$\theta_{ij}=\{\begin{array}{cc} \epsilon & \;\text{if}\;{R(o_{t}|s_{t})}_{ij}=0\\ \delta \;{R(o_{t}|s_{t})}_{ij} & \;\text{otherwise}\; \end{array}$$ (37)

We ran simulations for nine equally spaced values of caregiver responsiveness *q* ∈ {0.1, 0.2,…, 0.9}, for *h* ∈ {0.05, 0.5, 1, 1.5, 1.95}, *g* = 2, and varying value-pairs of (*m*, *n*). Recall that, under game theoretic assumptions, the emergence of the three organised attachment types as optimal responses to a caregiver with responsiveness *q* depends on the value of *h* relative to *gn*/*m* [66]. Here, we chose values of *n* such that, for *m* increasing in equal increments of 0.4 (from 0.1 to 3.3), (*m*, *n*) pairs have values of *gn*/*m* that increase in increments of 0.2 (from 0.1 to 1.7). These values provide a fairly comprehensive exploration of different reference configurations, under different levels of caregiver responsiveness.

**Fig 1:**
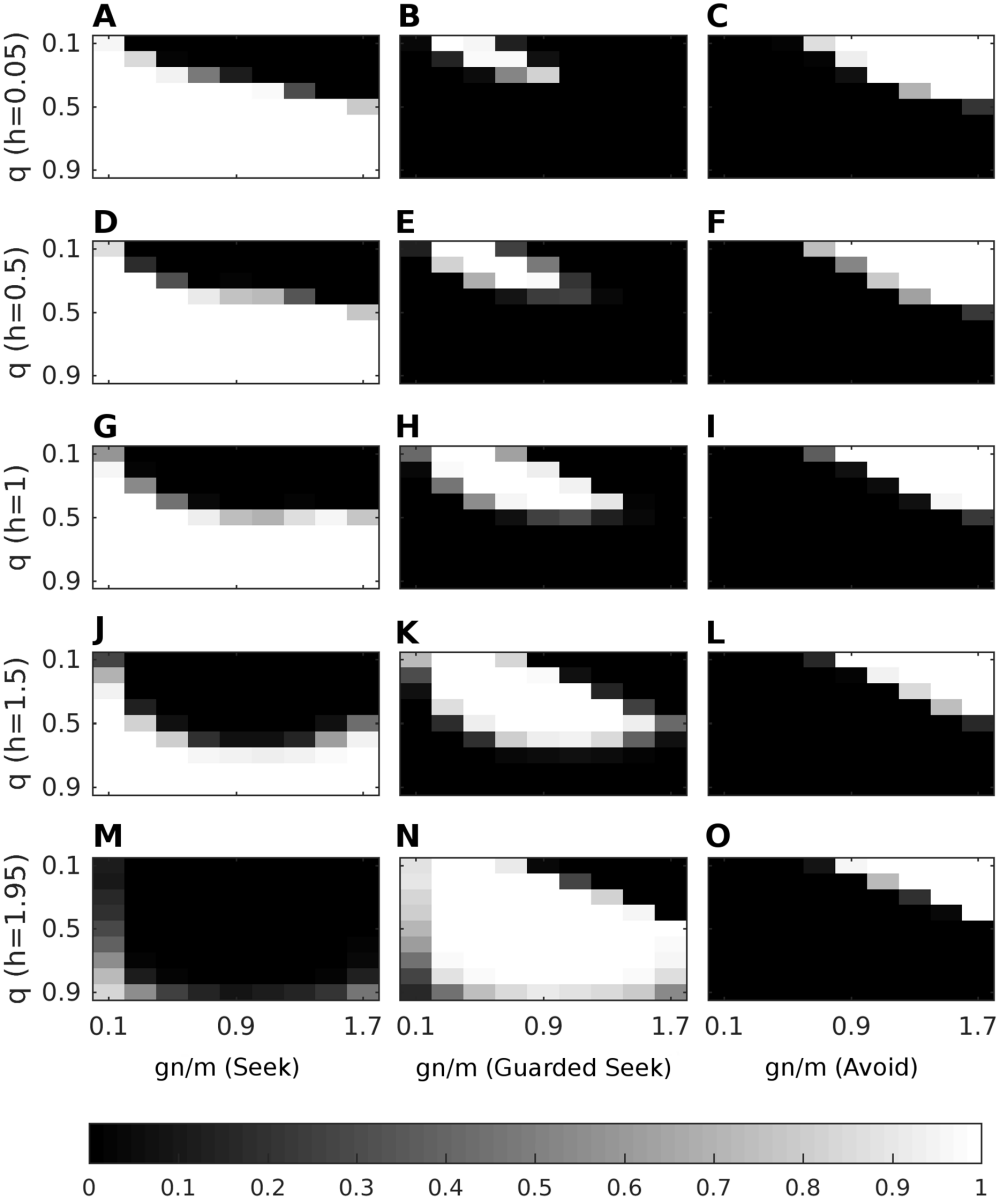
Heatmap of action selection, for various parameter configurations and a perfect generative model. Action selection proportions (black = 0, white = 1) per 4-step iteration of free energy minimisation (averaged over repetitions) for *g* = 2 and varying *h* (rows), *m* and *n* (x-axis) and *q* (y-axis). A: Proportions for Seek with *h* = 0.05. B: Proportions for Guarded Seek with *h* = 0.05. C: Proportions for Avoid with *h* = 0.05. D: Proportions for Seek with *h* = 0.5. E: Proportions for Guarded Seek with *h* = 0.5. F: Proportions for Avoid with *h* = 0.5. G: Proportions for Seek with *h* = 1. H: Proportions for Guarded Seek with *h* = 1. I: Proportions for Avoid with *h* = 1. J: Proportions for Seek with *h* = 1.5. K: Proportions for Guarded Seek with *h* = 1.5. L: Proportions for Avoid with *h* = 1.5. M: Proportions for Seek with *h* = 1.95. N: Proportions for Guarded Seek with *h* = 1.95. O: Proportions for Avoid with *h* = 1.95.

**Fig 2:**
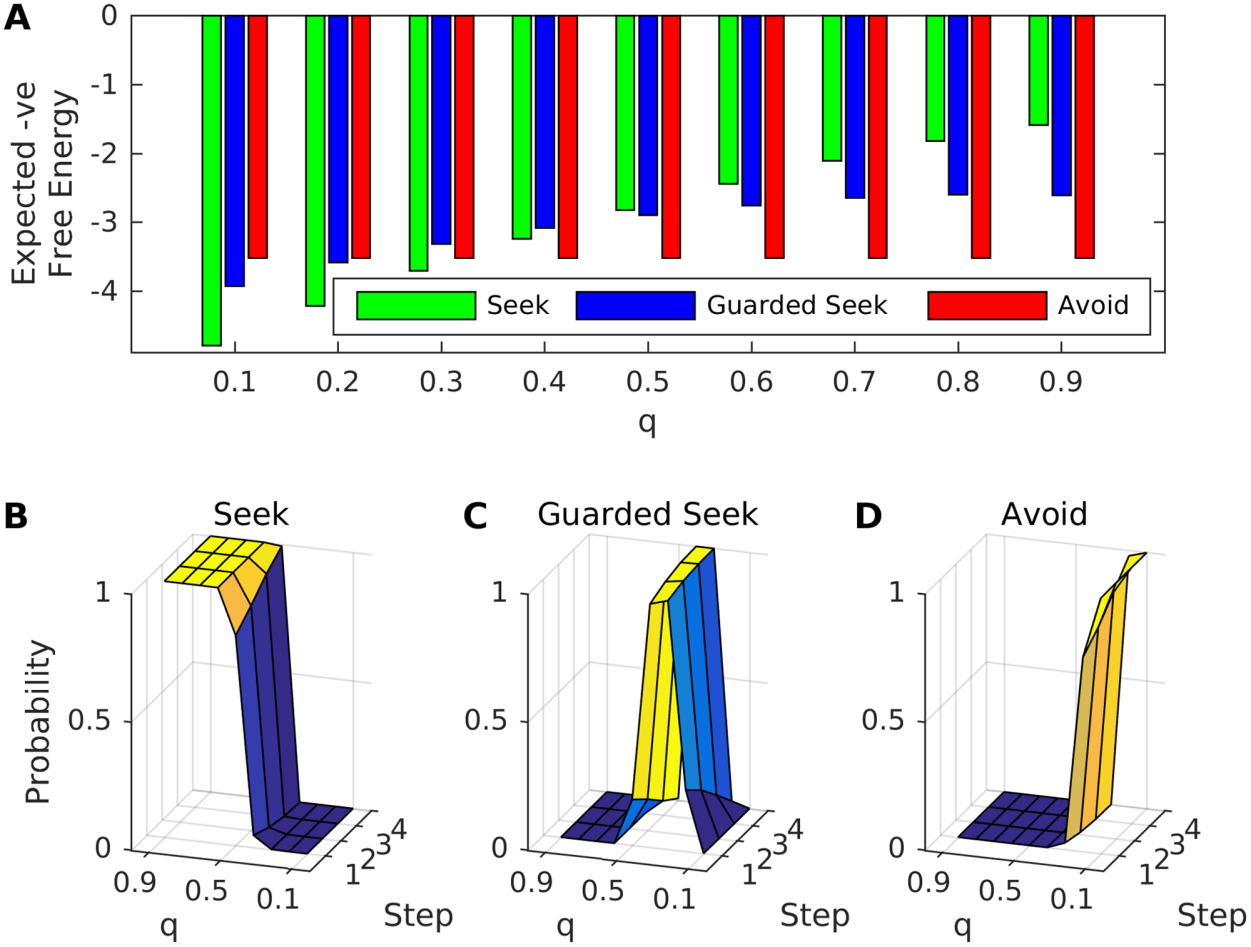
Expected negative free energies and action selection probabilities for perfect generative models. The charts show expected negative free energies and action selection probabilities for an agent that has a perfect generative model of their environment (which is defined by parameters *g* = 2, *h* = 0.75, *m* = 2 and *n* = 0.9, and varying responsiveness *q*). A: Mean (over repetitions) expected negative free energies for Seek, Guarded Seek and Avoid on final step of free energy minimisation (y-axis), for different values of q (x-axis). B: Mean (over repetitions) action selection probabilities (z-axis) on each of the 4 steps of free energy minimisation (x-axis) for different values of q (y-axis) for Seek (C: Guarded Seek, D: Avoid).

The results of this analysis showed that there are many parameter configurations for which highly consistent sequential selection of the three actions (corresponding to the three organised attachment types) emerges as *q* changes (Fig 1). One such parameter configuration is *g* = 2, *h* = 0.75, *m* = 2 and *n* = 0.9 (Fig 2), which we use in all simulations that follow. This configuration induces consistent Seek behaviour for high values of *q* approaching *q* = 0.9 (secure attachment), Guarded Seek behaviour for 0.3 ≤ *q* ≤ 0.4 (ambivalent attachment), and Avoid behaviour values approaching *q* = 0.1 (avoidant attachment). The precision parameter *α* controls the gradient of the curves and thus the extent to which attachment is organised in each case, such that increasing *α* (i.e. increasing prior expected precision) increases the extent to which these actions are chosen in each corresponding range of caregiver responsiveness.

Hitherto, we have considered how infants behave when they minimise free energy over interoceptive observations according to a generative model that perfectly encapsulates the probability that the caregiver will attentively respond to their attachment needs. In reality, however, infants are not born knowing the characteristics of their caregiver, but must instead learn this iteratively over repeated attachment interactions. Thus, we now consider an infant who learns the parameters of their generative model (with updates that minimise free energy), in order to see how the infant’s model and preferred behavioural policies might adapt accordingly as experience accrues. We pair this same infant with three different environments (i.e. prototypes of caregivers): highly responsive (*q* = 0.9), which corresponds to an attentive caregiver that mostly attends to the infant’s requests for attachment interaction; inconsistently responsive (*q* = 0.4), which corresponds to an erratic caregiver; and unresponsive (*q* = 0.1) which corresponds to a negligent caregiver that generally ignores the infant’s requests for attachment comfort. This allowed us to ascertain whether distinct, organised forms of attachment emerge.

We assume that each infant starts with a generative model with prior distributions that are uninformative with respect to caregiving behaviour. The prior distribution over hidden state transitions is thus now given by the following Dirichlet prior concentration parameters:

$${\phi (U_{w}\in U)}_{ij}=\{\begin{array}{cc} 1 & \;\text{if}\;2w-1\leq i\leq 2w\\ \epsilon & \;\text{otherwise}\; \end{array}$$ (38)

Similarly, we assume that the initial hidden state distribution is also uniform with respect to caregiving behaviour (and also initial state):

$$\xi ={\left\{1\right\}}^{J\times 1}$$ (39)

Since they are uniform with respect to caregiving behaviour, the parameters *ϕ* and *ξ* result in prior initial hidden state and hidden state transition distributions equivalent to the (uncertain) expectation of a caregiver with responsiveness *q* = 0.5, which (as we have seen previously) induces Seek behaviour in the infant for the preference and precision parameters identified above. The fact that these priors (flat with respect to responsiveness) result in an initial tendency in the infant towards Seek behaviour is consistent with the tenets of attachment theory; in that although infants are assumed to have no prior knowledge with respect to the effectiveness of their particular caregiver as an attachment figure, they are nonetheless (genetically) predisposed to seek out an attachment relationship with them. An innate attachment motivation appears to be present even in infants with autistic phenotypes, who display typical proximity seeking behaviour towards a caregiver under stressful situations despite broader impairments in social motivation [102, 103].

As in the previous simulations, we assume that the infant’s generative model of outcomes given hidden states is accurate (Eq 36) so that, given a hidden state (comprising the interaction between control states and caregiving responsiveness), the infant knows the corresponding outcome (comprising the interaction between control states and interoceptive outcomes) with certainty. Collectively, these priors represent the infant’s prior knowledge that, under states in which their attachment system is activated, seeking out the caregiver might result in either an increase or decrease in their stress level relative to the previous timestep (and that this increase or decrease can be reduced in magnitude with guarded or resistant behaviour). Conversely, avoidance of the caregiver will result in no (externally induced) change to their stress level. In other words, we assume prior knowledge in the infant that the caregiver can affect their internal states (and that they have the capacity to modulate this effect), but no specific knowledge regarding what the nature of this impact is likely to be.

Since we are primarily concerned with the learning of contingencies or transitions among hidden states (which entails caregiving responsiveness), in the simulations that follow we do not enable learning of observation model parameters (although the results below also generalise to the case in which observation model parameters are updated, given sufficiently large concentration parameter priors). We consider infants that start with the same prior parameters, but differ with respect to the type of caregiver that they are exposed to (highly responsive, inconsistent, or unresponsive). All results are averaged over 100 repetitions of 500 iterations (where each iteration comprises an episode of learning over four time steps).

**Fig 3:**
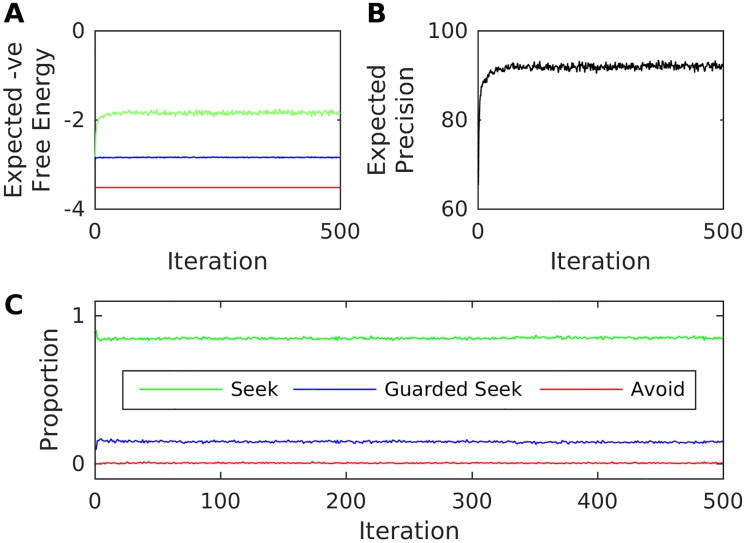
Secure attachment as free energy minimisation over interoceptive observations. The figure illustrates the emergence of secure attachment for an infant paired with a high-q (responsive) caregiver. A: Mean (over repetitions) expected negative free-energies for the three actions on the final step of each iteration. B: Mean (over repetitions) expected precision on the final-step of each episode. C: Mean (over repetitions) proportion each action was chosen over all iterations.

We begin by considering an environment for which *q* = 0.9, i.e. a caregiver who will (with relatively high probability) attend to the infant during high-stress states in which their attachment system is activated (Fig 3). Since the infant’s expectations about hidden states are initially flat with respect to caregiver behaviour, they have an initial tendency to Seek out the caregiver during these high stress states, with this preference sustained over iterations. The mean number of distinct actions chosen per iteration (a measure of organisation with respect to attachment behavioural strategy) settles at approximately 1.3. This suggests that (on average) these synthetic infants prefer sequentially-consistent Seek behaviour. Concomitantly, the expected precision rises as the infant comes to learn a more accurate generative model (and increasingly associates Seek behaviour with attainment of preferred interoceptive observations). This sort of infant comes to learn fairly accurate hidden state transition distributions for the Seek and Guarded Seek actions, but not for Avoid. This is because the interoceptive outcomes under the Avoid control state—the same for both caregiving Attend and Ignore behaviour—are seldom seen; however, this does not preclude the emergence of organised secure attachment.

**Fig 4:**
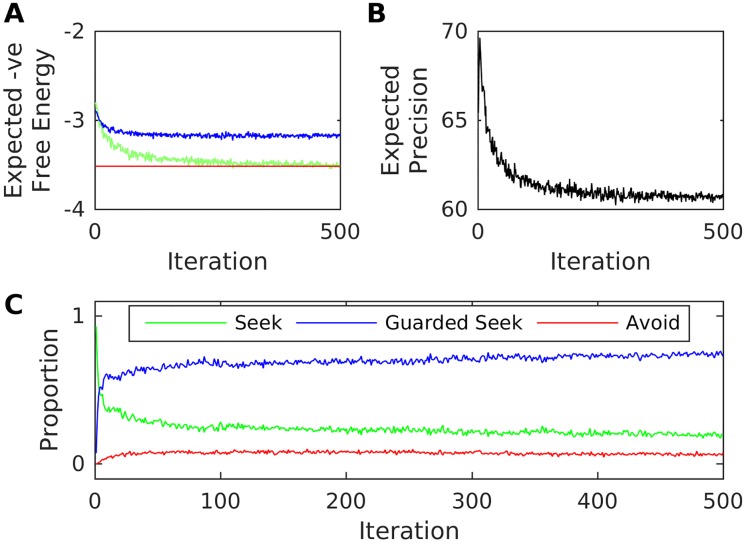
Ambivalent attachment as free energy minimisation over interoceptive observations. The figure illustrates the emergence of ambivalent attachment for an infant paired with a mid-q (inconsistent) caregiver. A: Mean (over repetitions) expected negative free-energies on the final step of each iteration. B: Mean (over repetitions) final-step expected precision. C: Mean (over repetitions) proportion each action was chosen over all iterations.

We now consider the same infant paired with an inconsistent caregiver (with *q* = 0.4), for which we previously demonstrated a strong tendency towards organised Guarded Seek (i.e. ambivalent) behaviour, when the infant knows about the caregiver. As above, the infant shows an initial tendency to Seek out the caregiver during high stress states. However, this initial Seek behaviour leads to the preferred interoceptive observation (highest stress reduction) with lower probability than the prior model suggests; resulting in exploratory switching of control behaviour and a fall in expected precision. As the transition model is accordingly updated, the infant increasingly comes to prefer policies involving sequential Guarded Seek behaviour—and this ambivalence becomes more organised as iterations progress (Fig 4). This infant learns a relatively accurate hidden state transition matrix, except under states involving Avoid (for the same reason as the secure infant above).

**Fig 5:**
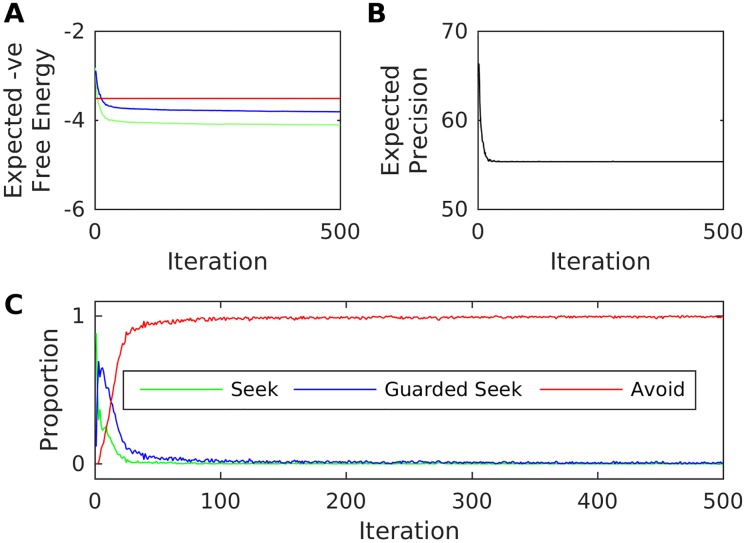
Avoidant attachment as free energy minimisation over interoceptive observations. The figure illustrates the emergence of avoidant attachment for an infant paired with a low-q (unresponsive) caregiver. A: Mean (over repetitions) expected negative free-energies on the final step of each iteration. B: Mean (over repetitions) final-step expected precision. C: Mean (over repetitions) proportion each action was chosen over all iterations.

Finally, we consider a consistently unresponsive caregiver (with *q* = 0.1), for which we previously showed a strong tendency towards organised Avoid behaviour. Again, due to their flat prior beliefs about state distributions, the infant has an initial tendency to Seek out the caregiver; however (as they learn that the caregiver is less responsive than their priors suggest) expected precision falls and the infant quickly come to prefer policies with predominantly Avoid actions; with this avoidance becoming highly consistent and organised (Fig 5). We note that this transition from preference for policies involving Seek behaviour to policies involving Avoid behaviour tends to occur via a period during which Guarded Seek behaviour emerges then disappears: this is a model prediction that can be tested empirically. As for the secure and ambivalent infants above, this infant learns a relatively accurate hidden state transition matrix for control states and (differentiable) transitions experienced.

Here, we have only considered convergence to secure and avoidant forms of attachment for *q* = 0.9 and *q* = 0.1 respectively, for prior hidden state concentration parameters of 1. Convergence to these organised forms of attachment can also occur for lower/higher values of *q* (e.g. *q* = 0.7 for secure and *q* = 0.2 for avoidant; according to the ranges outlined in the previous section). However, this convergence is more organised for these less extreme values when the prior concentration parameters are increased (which decreases the implicit learning rate, and increases the amount of initial exploration of control behaviour in the avoidant case).

### Exteroceptive observations, ambivalence and disorganisation

In the preceding simulations, we considered an infant who behaves, perceives and learns to minimise free energy based on interoceptive observations relating to changes in internal stress. We found that active inference over these interoceptive observations is sufficient for the emergence of behaviour resembling the organised attachment types, in infants who differ only with respect to the responsiveness *q* of the caregiver that they interact with. However, exteroceptive observations are also an important factor in infant attachment interactions: as noted by Bowlby, mother-infant attachment communications “are accompanied by the strongest of feelings and emotions”, which manifest in a variety of ways including facial expression, posture or tone of voice [1] and serve as “nonverbal communication of basic but very powerful attitudes in mind and potential action” [104, p.168].

As noted above, disrupted (atypical) affective communication according to the AMBIANCE scale [45, 46, Appendix G] has been linked to caregivers of both ambivalent and disorganised infants. Here, we focus on the ACE dimension of this scale, which has been highlighted in relation to the development of these two attachment types. The ACE dimension assesses the quality of communication (encompassing verbal communication, along with emotional communication in the form of tone of voice, facial expressions, gestures and mood presentation) between the infant and caregiver. More specifically, it captures atypical patterns of communication in terms of the congruence between infant-directed signals and subsequent behaviour; along with the nature of this behaviour. This has been conceptualised in terms of marked contingent mirroring in response to distress states expressed by the infant [105].

Thus, in addition to interoceptive observations, we now consider exteroceptive observations representing emotional cues from the caregiver. In particular, we consider how cues that conform to an infant’s prior beliefs, with respect to subsequent caregiving behaviour, might have an organising effect for either highly responsive (leading to secure) or unresponsive (avoidant) caregivers. On the other hand, we show that caregivers who provide cues that are either ambiguous or misleading, with respect to subsequent behaviour, might lead to ambivalent attachment: particularly when the caregiver inconsistently modulates stress. Finally, we show how caregivers who consistently increase infant stress when the infant seeks proximity but provide misleading cues before doing so, could have a disorganising effect on infant attachment formation.

#### A generative model for ambivalent attachment

The ACE dimension of AMBIANCE reflects caregiver communication that is misleading or ambiguous (contradictory) with respect to subsequent infant-directed behaviour (see [46, Appendix G] for further details). We will focus on these misleading and ambiguous cues here. Accordingly, we extend the hidden state transitions to incorporate caregiving status over to subsequent exchanges; i.e.,

$$\begin{array}{c} \begin{array}{cc} S & =U⊗Y:U\in \{Seek,Guarded\;Seek,Avoid\}\\ Y & =X_{t-1}⊗X_{t}:X\in \left\{Attend,Ignore\right\} \end{array} \end{array}$$ (40)

This means that we now have 3 × 2 × 2 = 12 hidden states, comprising all infant control states times all pairs of current and subsequent caregiving behaviour (we will refer to these as pairwise hidden state representations). The hidden state transition process now becomes:

$$G\left(\Omega_{w}\in \Omega \right)=M\left(0,w,L\right)⊗[\begin{array}{cccc} q & 0 & q & 0\\ \left(1-q\right) & 0 & \left(1-q\right) & 0\\ 0 & q & 0 & q\\ 0 & \left(1-q\right) & 0 & \left(1-q\right) \end{array}]$$ (41)

where:

$$M{\left(x,w,L\right)}_{ij}\in R^{L\times L}=\{\begin{array}{cc} 1 & \;\text{if}\;i=w\\ x & \;\text{otherwise}\; \end{array}$$ (42)

with hidden states indexed incrementally in rows and columns (as before). As previously, the prior parameters for the initial hidden state (Eq 39) and transition model are assumed to be uniform with respect to caregiving behaviour (where *J* and *L* are the appropriate dimensions):

$$\phi \left(U_{w}\in U\right)=M\left(\epsilon ,w,L\right)⊗{\left\{1\right\}}^{J/L\times J/L}$$ (43)

To model misleading or ambiguous ACE communications, we consider three different exteroceptive cues from the caregiver: a cue that the infant associates with subsequent attention, which is accurately delivered by the caregiver with probability *a*; no cue, which the infant associates with subsequent inattention and which is accurately delivered with probability *b*; and an ambiguous cue, that the infant associates with *both* subsequent Attend and Ignore behaviour and which the caregiver gives with probability *c*. The total observation set is now the product of the infant’s interoceptive observations (relating to internal stress levels, as before) and these additional exteroceptive cues from the caregiver (see S1 Appendix for details).

In addition to the prior associations between interoceptive observations and hidden states discussed in the previous section; this model therefore encodes prior beliefs associating a particular exteroceptive cue with subsequent caregiving Attend behaviour, another (lack of) exteroceptive cue with subsequent Ignore behaviour, and a third (ambiguous) cue which is expected under hidden states in which the subsequent behaviour is either Attend or Ignore. As before, Attend and Ignore refer to caregiving behaviours (or lack thereof) that effectively regulate the infant’s internal state, rather than to interaction (or lack of interaction) more broadly. Thus, we assume that these exteroceptive cues can be delivered by the caregiver in both Attend and Ignore states. For example, the caregiver might ‘ignore’ the infant (e.g. not be providing soothing physical contact to regulate their internal state) but indicate in terms of a cue (e.g., inviting speech) that they intend to ‘attend’ to their needs in the subsequent time step. Conversely, the caregiver might ‘attend’ to the infant’s attachment needs, but adopt an emotionless (no cue) or ambiguous facial expression while doing so. We furthermore assume that the infant will always observe ‘no cue’ when they Avoid. As for the case in which we considered only interoceptive observations, we assume that the infant’s likelihood model of observations given hidden states has been learned. This allows us to focus on the learning of proximity seeking contingencies or hidden state transitions.

#### Simulations

We begin by considering the three organised (secure, avoidant, ambivalent) forms of attachment. As discussed above, empirical evidence suggests elevated rates of ACEs in caregivers of ambivalent infants compared to caregivers of secure and avoidant infants. We therefore ran simulations for an infant paired with four types of caregiver: a highly responsive caregiver who signals subsequent behaviour appropriately and unambiguously (*q* = 0.9, *a* = *b* = 1, *c* = 0); a highly unresponsive caregiver who signals subsequent behaviour appropriately and unambiguously (*q* = 0.05, *a* = *b* = 1, *c* = 0); an inconsistent caregiver who signals subsequent regulatory behaviour appropriately and unambiguously (*q* = 0.4, *a* = *b* = 1, *c* = 0); and an inconsistent caregiver who commits affective communication errors (*q* = 0.4, varying values for *a*, *b*, *c*). All the ensuing results were averaged over 100 repetitions of 1000 iterations (as above, an iteration corresponds to four time steps or state transitions).

**Fig 6:**
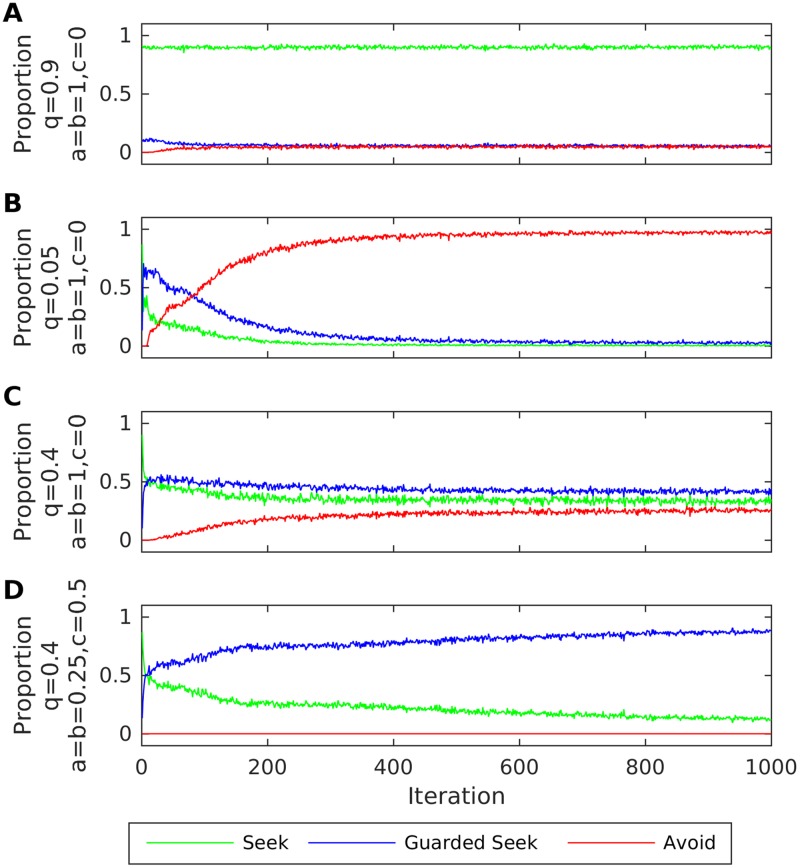
Secure, avoidant and ambivalent attachment with additional exteroceptive cues. This figure illustrates the mean (over repetitions) proportion of actions selected. A: Proportions for an infant paired with a highly responsive caregiver (q = 0.9) with no ACEs (a = b = 1, c = 0). B: Proportions for an infant paired with a highly unresponsive caregiver (q = 0.05) with no ACEs (a = b = 1, c = 0). C: Proportions for an infant paired with an inconsistent caregiver (q = 0.4) with no ACEs (a = b = 1, c = 0). D: Proportions for an infant paired with an inconsistent caregiver (q = 0.4) with both ambiguous and misleading ACEs (a = b = 0.25, c = 0.5).

Fig 6 shows the average proportion of actions selected along with mean number of actions chosen per iteration for infants paired with these varieties of caregiving. Crucially, a lack of ACEs in highly responsive and unresponsive caregivers results in organised secure and avoidant attachment, respectively. On the other hand, an infant paired with an inconsistent caregiver—who signals subsequent behaviour appropriately—does not express an organised form of attachment; however, when this inconsistent caregiver provides misleading (*a* = *b* = 0.25) and ambiguous (*c* = 0.5) cues, a relatively organised form of ambivalent attachment emerges. In other words, consistent with empirical observations, ACEs can have an organising effect in infants of highly inconsistent caregivers.

**Fig 7:**
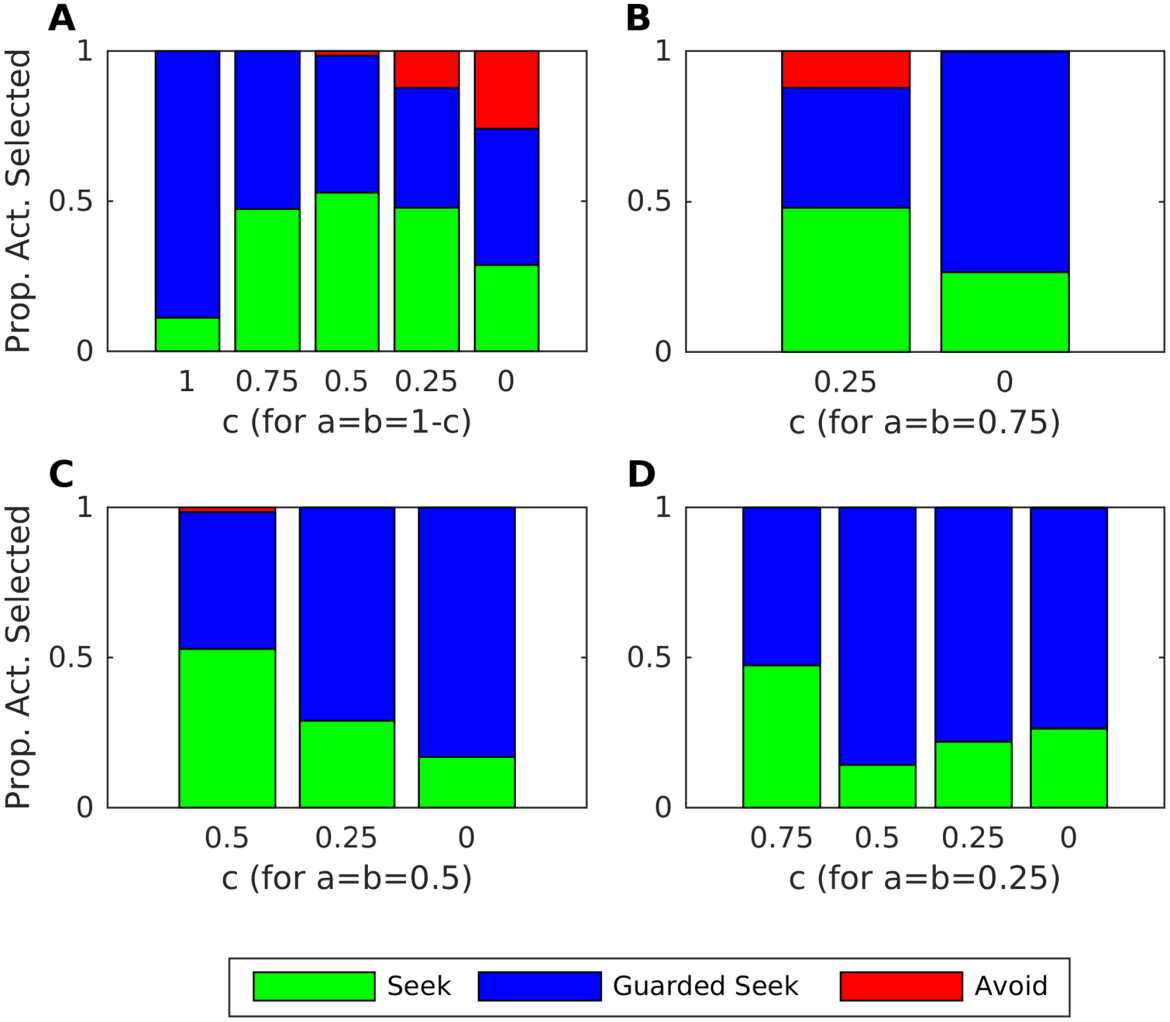
Ambivalent attachment for combinations of misleading and ambiguous exteroceptive cues. Mean (over repetitions) proportion of actions chosen by the infant during the last 10 iterations, when they were paired with an inconsistent caregiver (q = 0.4) exhibiting varying rates and types of affective communication errors. A: Proportions for a = b = 1-c and c decreasing from 1 to 0. B: Proportions for a = b = 0.75 with c decreasing from 0.25 to 0. C: Proportions for a = b = 0.5 and c decreasing from 0.5 to 0. D: Proportions for a = b = 0.25 and c decreasing from 0.75 to 0.

Fig 7 shows the mean proportion of actions chosen during the last 10 iterations by an infant paired with an inconsistent caregiver (*q* = 0.4) for varying values of *a*, *b*, *c*. The simulation results show how various combinations of misleading and ambiguous ACEs can result in relatively organised forms of ambivalent attachment, compared to the case where cues are accurate and unambiguous (*a* = *b* = 1, *c* = 0). Ambivalent attachment is particularly organised for three of the configurations we examined: when all cues are ambiguous (*a* = *b* = 0, *c* = 1), when cues are either misleading or accurate with equal probability (*a* = *b* = 0.5, *c* = 0), and for a mixture of misleading and ambiguous cues (*a* = *b* = 0.25, *c* = 0.5).

We now consider ACEs and the formation of disorganised attachment. Lyons-Ruth et al. found that particular ACE items were three times more prevalent in mothers of disorganised infants [44]. One of these items was “inviting approach verbally but then distancing”; a misleading cue that corresponds to *b* < 1 in the generative process. Although caregivers of disorganised infants (who are also prone to withdrawal and frightening behaviours) are typically thought to increase infant stress levels at a greater magnitude than caregivers of avoidant infants (who instead simply ignore when the infant seeks proximity), we note that for *a* = *b* = 1 and *c* = 0 the infants of such caregivers would learn predominantly Avoid behaviour (as in the case of the avoidant infant examined above). Thus, for simplicity, we consider the same interoceptive preferences as before, to show how this particular ACE (*b* < 1) can have a disorganising effect; namely the disorganising effect of caregivers who increase the infant’s stress when they seek proximity.

**Fig 8:**
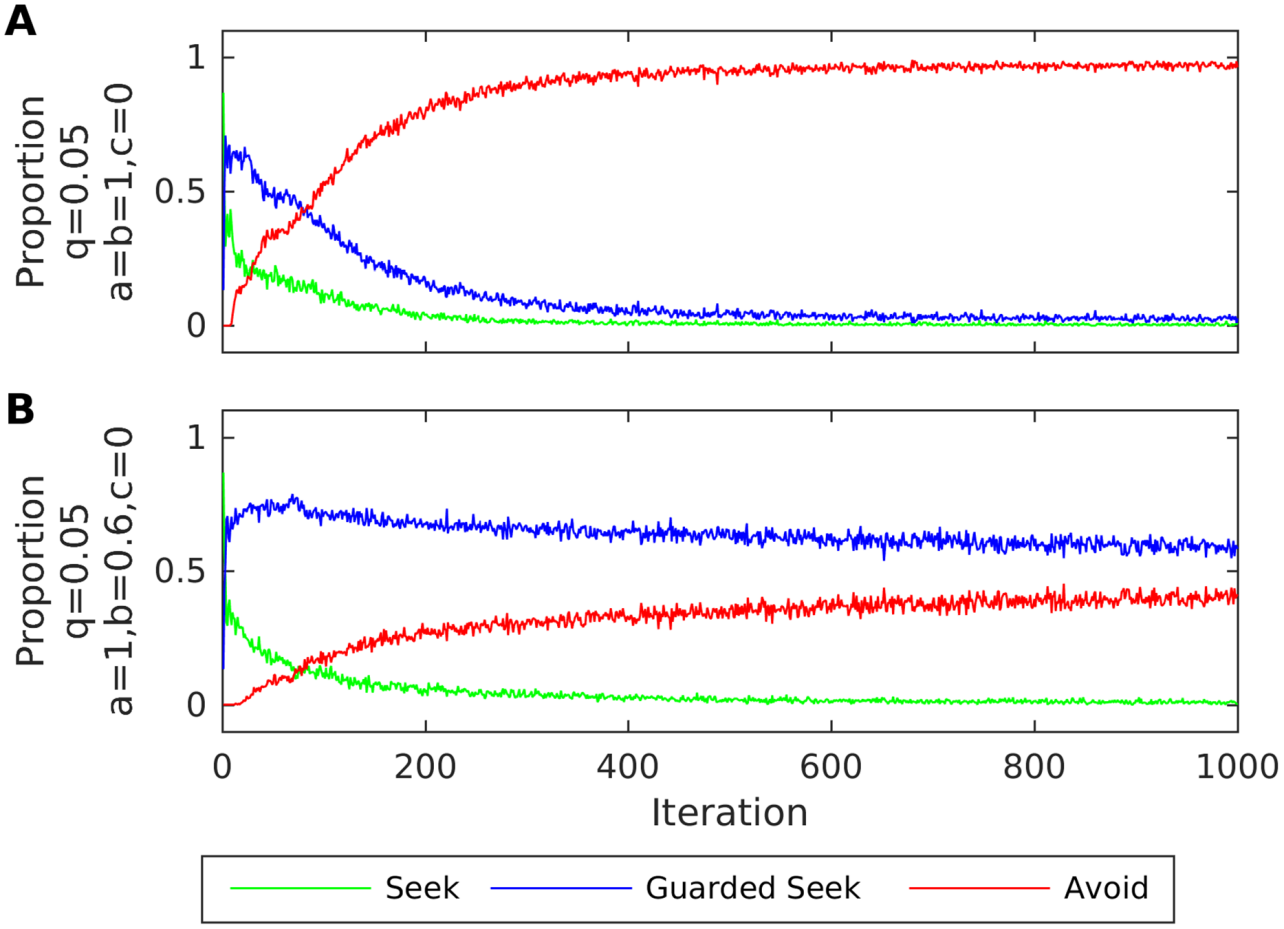
Avoidant and disorganised attachment with exteroceptive cues. Mean (over repetitions) proportion each action was chosen over all iterations. A: Proportions for an infant paired with a highly unresponsive caregiver (q = 0.05) with no ACEs (a = b = 1, c = 0). B: Proportions for an infant paired with a highly unresponsive caregiver (q = 0.05) with misleading ACEs about subsequent Ignore behaviour (a = 1, b = 0.6, c = 0).

**Fig 9:**
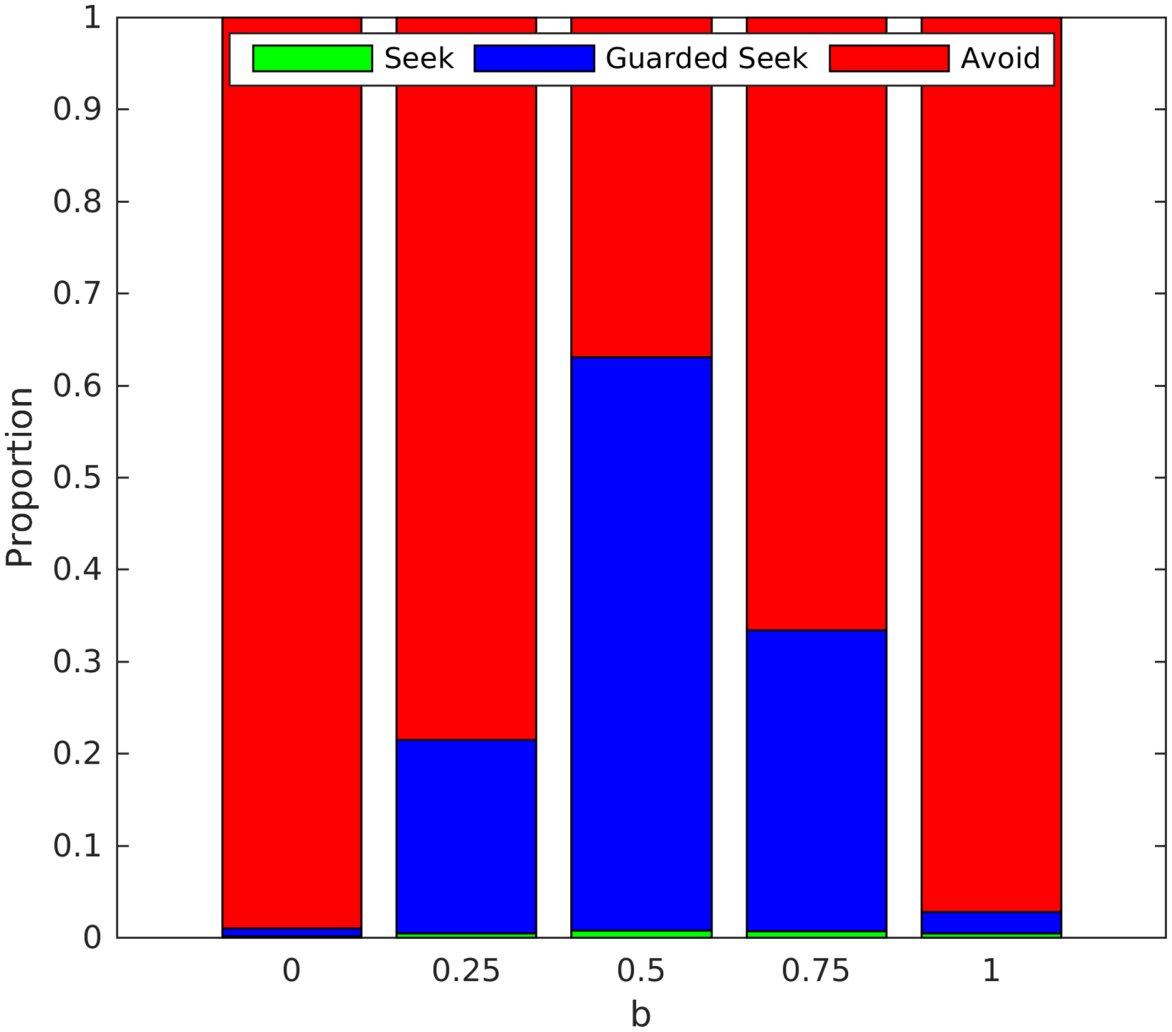
Disorganised attachment and misleading exteroceptive cues. Mean (over repetitions) proportion each action was chosen by the infant during the last 10 iterations (y-axis), when they were paired with a highly unresponsive caregiver (q = 0.05) exhibiting varying rates of misleading affective communication errors (values of *b* ∈ {0, 0.25, 0.5, 0.75, 1} along the x-axis, for fixed *a* = 1 and *c* = 0).

Fig 8 shows the mean proportion of selected actions and mean number of actions chosen per iteration for infants paired with caregivers who, with high probability (*q* = 0.05), increase stress when they seek proximity. In accordance with empirical studies, while caregivers who signal all subsequent behaviour accurately (*a* = *b* = 1, *c* = 0) lead infants towards organised avoidant attachment, caregivers that signal subsequent Attend behaviour accurately but subsequent Ignore behaviour inaccurately with probability 60% (*a* = 1, *b* = 0.6, *c* = 0) have a highly disorganising effect on infant behaviour. The mean proportion each action was chosen during the last 10 iterations by an infant paired with caregivers with *q* = 0.05 and varying values of *b* (with *a* = 1 and *c* = 0 in all cases) is shown in Fig 9.

To understand in detail why the infant of the small-*q* caregiver who exhibits ACEs chooses roughly equal proportions of Avoid and Guarded Seek behaviour within each episode, it is necessary to understand the role of expected free energy in policy selection. In [85] it was shown that the expected (negative) free energy or ‘quality’ of policy *π* at time *τ* > *t* can be decomposed into pragmatic (extrinsic) and epistemic (intrinsic) terms:

$$Q_{\tau }\left(\pi \right)=E_{Q(o_{\tau }|\pi )}[\text{ln}\;P\left(o_{\tau }\right)+KL[Q\left(s_{\tau }|o_{\tau },\pi \right)||Q\left(s_{\tau }|\pi \right)]]$$ (44)

The extrinsic component $E_{Q(o_{\tau }|\pi )}[\text{ln}\;P(o_{\tau })]$ is the utility of outcomes (expected under the predictive posterior distribution) defined in terms of prior preferences. In our model, extrinsic value corresponds to the preference the agent has for a particular interoceptive outcome (stress increase or reduction), since they are assumed to be indifferent with respect to exteroceptive observations. The epistemic value $E_{Q(o_{\tau }|\pi )}[KL[Q\left(s_{\tau }|o_{\tau },\pi \right)||Q\left(s_{\tau }|\pi \right)]$ quantifies the reduction in uncertainty about hidden states based on the outcome. Epistemic value drives exploration of control behaviour, in the sense that an agent can select policies predicting outcomes with relatively low extrinsic value if these outcomes reduce uncertainty with respect to hidden states. Epistemic value is an expected KL divergence or information gain that endows a particular policy with salience or epistemic affordance.

**Fig 10:**
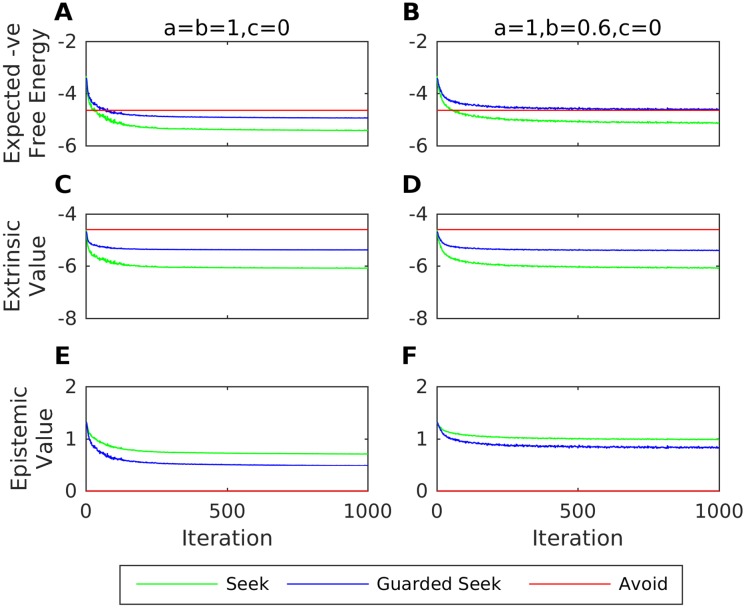
Extrinsic and epistemic value for avoidant and disorganised attachment with exteroceptive cues. A: Mean (over repetitions) expected negative free energies for each action on the final step of each iteration, for infants interacting with a low-q (unresponsive) caregiver displaying no ACEs, i.e. *a* = *b* = 1 and *c* = 0. B: Mean (over repetitions) expected negative free energies for infants interacting with a low-q (unresponsive) caregiver displaying ACEs with *a* = 1, *b* = 0.6 and *c* = 0. C: Mean (over repetitions) extrinsic values for infants interacting with a low-q (unresponsive) caregiver displaying no ACEs, i.e. *a* = *b* = 1 and *c* = 0. D: Mean (over repetitions) extrinsic values for infants interacting with a low-q (unresponsive) caregiver displaying ACEs with *a* = 1, *b* = 0.6 and *c* = 0. E: Mean (over repetitions) epistemic values for infants interacting with a low-q (unresponsive) caregiver displaying no ACEs, i.e. *a* = *b* = 1 and *c* = 0. F: Mean (over repetitions) epistemic values for infants interacting with a low-q (unresponsive) caregiver displaying ACEs with *a* = 1, *b* = 0.6 and *c* = 0.

Fig 10 shows the expected negative free energy, and extrinsic and epistemic value for each action on the final step of each iteration (shown as the mean over repetitions), for both avoidant and disorganised infants. Both types of infant come to predict similar interoceptive outcomes for Seek and Guarded Seek behaviours (corresponding to the caregiver Ignoring them on the final exchange) and thus assign similar extrinsic value to these actions. However, epistemic value for Seek and Guarded Seek actions remains relatively high for the disorganised compared to the avoidant infant over iterations. The avoidant infant accrues meaningful information with respect to pairwise hidden state transitions, whereas (as a result of experiencing ACEs) the disorganised infants do not: these infants come to predict future hidden states involving mixed pairwise Attend and Ignore behaviour. The epistemic value of trying to resolve the consequent ambiguity is sufficient to give rise to disorganised behaviour.

## Discussion

Building on an established decision theoretic model of organised attachment, we have proposed a formulation of the emergence of infant attachment in terms of free energy minimisation (i.e., active inference). In particular, we considered infant agents that minimise free energy over interoceptive outcomes associated with changes in their internal stress levels. Using this model, we demonstrated how an infant with initially uninformative (flat) beliefs about hidden state transitions—with respect to caregiving responsiveness—can come to acquire either a secure, avoidant or ambivalent form of organised attachment, with the type of attachment depending only on the responsiveness of the caregiver. Based on evidence relating to the commission of Affective Communication Errors (ACE) in caregivers—of both ambivalent and disorganised infants—we then extended this model to consider exteroceptive cues from the caregiver. We focused on one ACE that has been found to be three times more prevalent in caregivers of disorganised infants; a cue that inaccurately implies subsequent attention. Our simulations showed how such a misleading and uncertainty-inducing cue might have a disorganising effect in infants of caregivers who (with high probability) increase infant distress when they seek proximity. This disorganising effect was driven by a Bayes optimal imperative to reduce uncertainty; in other words select actions that have the greatest epistemic value or affordance. Since no particular ACE items on the AMBIANCE scale have as yet been associated with ambivalent forms of attachment, we explored the effect on the infant of both exteroceptive cues that are misleading and ambiguous with respect to subsequent caregiving behaviour. We showed how the introduction of various combinations of such ACEs might have an organising (towards ambivalence) effect in infants paired with these inconsistent caregivers. Our model makes a novel prediction that can potentially be tested empirically; namely, combinations (distributions) of misleading and ambiguous ACEs will lead to the organised forms of ambivalent attachment.

The functional anatomy of decision making in the context of free energy minimisation has been reviewed in [106] and [107], where the cerebellum is thought to play a key role in habit learning. In recent years, a consensus has emerged that most of the human cerebellum projects to cerebral association networks, thus playing a fundamental role in cognition as well as motor function [108]. It is also thought that the cerebellum might encode internal models that reproduce the essential properties of mental representations in the cerebral cortex [109]. This view is consistent with the assumption that attachment types might involve the cerebellum. On the other hand, there is also a wealth of evidence to implicate introception in cerebral hierarchies; particularly in the context of the interoceptive inference associated with stress and affiliative behaviours. See for example [110–112]. This would speak to the involvement of the amygdala, anterior insular and anterior cingulate cortices; not to mention the medial prefrontal cortex and basal ganglia [106, 113].

One potential application of models—such as the one presented here—is phenotyping of parent-infant dyads [114]. However, there are a number of ways that future iterations of the model could be improved. For example, we considered active inference over fixed-length episodes in which all hidden states were taken to be states in which the infant’s attachment system is active; i.e., we did not explicitly consider a return to baseline stress level for the infant. This was for reasons of simplicity, and also since evidence with respect to the time taken to return to baseline for avoidant, ambivalent and disorganised infants is currently either inconclusive (for interactions in which caregiver responsiveness is controlled) or unavailable (for uncontrolled interactions). As more empirical data becomes available, the model could be extended to include transitions from the hidden states considered above to an additional state (associated with a highest-preference interoceptive observation); representing deactivation of the infant’s attachment system, with stress change parameters, transition probabilities and caregiving responsiveness set in order to accommodate the empirical data for each of these distinct attachment types. Future work could also extend the scope of the model to capture the secure-base exploration paradigm more fully, to consider how undesirable stress states might arise during the course of environmental exploration and how exploration might resume on transition to this baseline state.

In accordance with studies that have found only the ACE dimension of the AMBIANCE scale to be a differentiator of disorganised compared to organised (secure and avoidant) attachment, and elevated ACEs in caregivers of ambivalent infants, we focused on the ACE dimension in our model. For disorganised attachment, we considered one particular type of ACE (cues that are misleading on subsequent caregiving inattention), whereas for ambivalent attachment we considered a number of distributions under which misleading and/or ambiguous cues were delivered with varying frequency. In particular, we considered these exteroceptive cues to be misleading or ambiguous to the infant a priori, captured using relatively large priors in the infant’s likelihood model of observations given hidden states. We made this modelling assumption because the AMBIANCE scale describes ACEs in terms of broad groups of emotional and/or verbal cues. We therefore focused on the learning of contingencies (i.e., state transitions that depend on caregiving responsiveness). Future work could consider how ambiguity in exteroceptive cues might arise as a result of learning (and the two models could be compared using Bayesian model comparison). In addition, elevated rates on other dimensions of the AMBIANCE disrupted affective communication scale have been associated with disorganised (withdrawal, disorientation, negative/intrusive and role confusion) and resistant (disorientation, negative/intrusive) forms of attachment. Future models could consider these other atypical caregiving behaviours, along with other cues described by the ACE dimension. An attempt to capture additional aspects of disorganised (such as dissociative-like freezing) and ambivalent (e.g. hyperactivation) infant attachment behaviour could also be made: in the case of the ambivalent infant, a self-induced increase in stress is believed to be a strategy to increase the likelihood of subsequently attentive caregiving, which can be captured relatively easily in the hidden state transition structure. The broad nature of behaviours described by the four attachment categorisations that we have considered here has led to various attempts to sub-categorise these attachment types. Thus, one might also attempt to differentiate between, for example, the ambivalent subtypes identified in [19] or the disorganised subtypes associated with the Hostile/Helpless caregiving profiles in [44]. As further extensions to the model, one might attempt to capture prolonged states of mind in the caregiver over each attachment episode (corresponding to contexts in the scenario modelled in [85]) and an infant agent who learns a hierarchical generative model in which higher levels contextualise lower levels [85, 115]. It would also be interesting to consider the subjective emotional experiences (defined in [116] in terms of the first and second-order time derivatives of free energy) of infants paired with distinct types of caregiver, particularly fear (in light of Main’s classical hypothesis linking this emotion with disorganisation).

Finally, a recent update to the discrete free energy minimisation framework used here additionally accounts for habit learning [107]. This would be interesting to consider within the context of attachment, along with interventions that are used to treat related conditions of severe psychopathology such as BPD (e.g. cognitive behavioural [117], schema [118] and mentalization [119] therapies) that may involve the overcoming of deeply ingrained attachment-related habits. One intervention in particular—that would be interesting to consider—is Self-Attachment therapy [77, 78]. Self-Attachment aims to redress suboptimal early attachment experience by way of creating a secure attachment relationship that is fully internalised within the individual, using techniques that are thought to induce oxytocin and dopamine-mediated plasticity in key attachment-related neural circuitry [79, 80]. It has been proposed that oxytocin plays a role in encoding the precision of interoceptive signals and therefore is involved in the association of interoceptive and exteroceptive observations within generative models of the self [120]. An interesting avenue for future work would thus be to formulate the hypothesised dynamics underlying a successful application of Self-Attachment therapy in terms of active inference.

## Supporting information

S1 Appendix Details for the model including exteroceptive observations. (PDF)
