# Supplementary material for: Intrinsic and extrinsic motivators of attachment under active inference
Source: PLoS One. 2018 Apr 5;13(4):e0193955. doi: 10.1371/journal.pone.0193955 (PMC5886414; doi:10.1371/journal.pone.0193955)
Supplement: S1 Appendix — (PDF) [file pone.0193955.s001.pdf]

S1 Appendix: Details for the model including exteroceptive observations

For the extended model including exteroceptive observations, the distribution of outcomes given hidden states in the generative process is:

$$R(o_t|s_t) = \begin{bmatrix} \lambda^{(1)} & & \\ & \lambda^{(2)} & \\ & & \lambda^{(3)} \end{bmatrix} \tag{1}$$

with:

$$\lambda^{(1)} = \begin{bmatrix} a & (1-b-c) & 0 & 0 \\ (1-a-c) & b & 0 & 0 \\ c & c & 0 & 0 \\ 0 & 0 & 0 & 0 \\ 0 & 0 & 0 & 0 \\ 0 & 0 & 0 & 0 \\ 0 & 0 & a & (1-b-c) \\ 0 & 0 & (1-a-c) & b \\ 0 & 0 & c & c \\ 0 & 0 & 0 & 0 \\ 0 & 0 & 0 & 0 \\ 0 & 0 & 0 & 0 \\ 0 & 0 & 0 & 0 \\ 0 & 0 & 0 & 0 \\ 0 & 0 & 0 & 0 \end{bmatrix}$$
$$\lambda^{(2)} = \begin{bmatrix} 0 & 0 & 0 & 0 \\ 0 & 0 & 0 & 0 \\ 0 & 0 & 0 & 0 \\ a & (1-b-c) & 0 & 0 \\ (1-a-c) & b & 0 & 0 \\ c & c & 0 & 0 \\ 0 & 0 & 0 & 0 \\ 0 & 0 & 0 & 0 \\ 0 & 0 & 0 & 0 \\ 0 & 0 & a & (1-b-c) \\ 0 & 0 & (1-a-c) & b \\ 0 & 0 & c & c \\ 0 & 0 & 0 & 0 \\ 0 & 0 & 0 & 0 \\ 0 & 0 & 0 & 0 \end{bmatrix}, \lambda^{(3)} = \begin{bmatrix} 0 & 0 & 0 & 0 \\ 0 & 0 & 0 & 0 \\ 0 & 0 & 0 & 0 \\ 0 & 0 & 0 & 0 \\ 0 & 0 & 0 & 0 \\ 0 & 0 & 0 & 0 \\ 0 & 0 & 0 & 0 \\ 0 & 0 & 0 & 0 \\ 0 & 0 & 0 & 0 \\ 0 & 0 & 0 & 0 \\ 0 & 0 & 0 & 0 \\ 0 & 0 & 0 & 0 \\ 0 & 0 & 0 & 0 \\ 1 & 1 & 1 & 1 \\ 0 & 0 & 0 & 0 \end{bmatrix} \tag{2}$$

where a, b, c take varied values as described in the main text. Observable outcomes are thus now the set of all tuples of control states (U, corresponding to the 3 resultant proximities), interoceptive observations (the 5 stress levels I) and exteroceptive observations (the 3 caregiving cues), giving a total of $W = 45$ observable outcomes. The probability of any particular observable outcome (rows of

R) given a hidden state (columns of R) is parametrised by a, b, c (with a parametrising observable outcomes involving exteroceptive cues that the infant associates with subsequent attention; b parametrising outcomes involving lack of a cue, that the infant associates with subsequent inattention; and c parametrising outcomes involving ambiguous cues, that the infant associates with both subsequent attention and inattention). Similarly, the infant's likelihood model of observations given hidden states is now given by:

$$\theta = \{\epsilon\}^{W \times J} + \begin{bmatrix} \theta^{(1)} & & \\ & \theta^{(2)} & \\ & & \theta^{(3)} \end{bmatrix} \tag{3}$$

with $\epsilon = 10^{-10}$ as before, $J = 12$ total hidden states, and:

$$\theta^{(1)} = \begin{bmatrix} 900 & 0 & 0 & 0 \\ 0 & 900 & 0 & 0 \\ 100 & 100 & 0 & 0 \\ 0 & 0 & 0 & 0 \\ 0 & 0 & 0 & 0 \\ 0 & 0 & 0 & 0 \\ 0 & 0 & 900 & 0 \\ 0 & 0 & 0 & 900 \\ 0 & 0 & 100 & 100 \\ 0 & 0 & 0 & 0 \\ 0 & 0 & 0 & 0 \\ 0 & 0 & 0 & 0 \\ 0 & 0 & 0 & 0 \\ 0 & 0 & 0 & 0 \\ 0 & 0 & 0 & 0 \end{bmatrix}, \theta^{(2)} = \begin{bmatrix} 0 & 0 & 0 & 0 \\ 0 & 0 & 0 & 0 \\ 0 & 0 & 0 & 0 \\ 900 & 0 & 0 & 0 \\ 0 & 900 & 0 & 0 \\ 100 & 100 & 0 & 0 \\ 0 & 0 & 0 & 0 \\ 0 & 0 & 0 & 0 \\ 0 & 0 & 0 & 0 \\ 0 & 0 & 900 & 0 \\ 0 & 0 & 0 & 900 \\ 0 & 0 & 100 & 100 \\ 0 & 0 & 0 & 0 \\ 0 & 0 & 0 & 0 \\ 0 & 0 & 0 & 0 \end{bmatrix} \tag{4}$$

$$\theta^{(3)} = \begin{bmatrix} 0 & 0 & 0 & 0 \\ 0 & 0 & 0 & 0 \\ 0 & 0 & 0 & 0 \\ 0 & 0 & 0 & 0 \\ 0 & 0 & 0 & 0 \\ 0 & 0 & 0 & 0 \\ 0 & 0 & 0 & 0 \\ 0 & 0 & 0 & 0 \\ 0 & 0 & 0 & 0 \\ 0 & 0 & 0 & 0 \\ 0 & 0 & 0 & 0 \\ 0 & 0 & 0 & 0 \\ 0 & 0 & 0 & 0 \\ 1000 & 1000 & 1000 & 1000 \\ 0 & 0 & 0 & 0 \end{bmatrix} \tag{5}$$
